# Novel Knotted Solenoid fold with order-shifted coil arrangement leads to nontrivial 3_1_ topology

**DOI:** 10.1073/pnas.2525920123

**Published:** 2026-04-22

**Authors:** Maciej Sikora, Mariusz Mozajew, Julia A. Sikorska, Fernando Bruno da Silva, Agata P. Perlinska, Anna Kluza, Szymon Niewieczerzal, Maciej Lukaszewicz, Beata Wielgus-Kutrowska, Karolina Stachurska-Korzeniowska, Sophie E. Jackson, Joanna I. Sulkowska

**Affiliations:** ^a^Centre of New Technologies, University of Warsaw, Warsaw 02-097, Poland; ^b^Division of Biophysics, Institute of Experimental Physics, Faculty of Physics, University of Warsaw, Warsaw 02-093, Poland; ^c^Yusuf Hamied Department of Chemistry, Cambridge CB2 1EW, United Kingdom

**Keywords:** structural biology, nontrivial topology, Knotted Solenoid fold, protein design, evolution

## Abstract

Herein, we present crystal structures of proteins adopting a fold not identified among known β-solenoids in current structural databases, a Knotted Solenoid. These proteins exhibit a characteristic solenoidal architecture, closely resembling β-solenoid proteins. However, a unique “skip-and-backtrack” shift is observed: One coil skips a rotation while the next realigns, distinguishing this fold from previously described solenoids and allowing the formation of a 3_1_ (trefoil) knot. Moreover, the proteins form homodimers, and conservation of interface residues suggests a shared oligomerization state across all Knotted Solenoids. This fold is exclusive to a specific group of bacteria and remains structurally conserved despite high sequence variability (pairwise identities down to 6%). Conserved residues are observed at the beginning of each coil and within the knot core, suggesting functional or structural significance, and an independent evolutionary path to unknotted solenoids. In vitro chemical and thermal stability studies showed fully reversible unfolding in urea, and no additional transitions even in high concentrations of guanidinium chloride. The far-ultraviolet circular dichroism unfolding kinetics showed relatively rapid unfolding. Explicit-solvent molecular dynamics simulations and a generative deep learning model show that topological constraints stabilize “skip-and-backtrack” shift in the Knotted Solenoid. A monomeric unit can self-tie into the native 3_1_ knotted state through a slipknot intermediate. It then interacts with another chain via its hydrophobic surface, promoting second chain folding and dimerization. The identification of novel knotted proteins within a previously considered unknotted fold provides an opportunity to investigate the evolutionary pressures and functional implications of knotting in shaping protein architecture.

## Significance

We present the crystal structures of proteins adopting the Knotted Solenoid fold, a solenoidal architecture uniquely capable of forming a 3_1_ knot through a “skip-and-backtrack” shift, challenging the assumption that all β-solenoid proteins are unknotted. Knotted Solenoids occur exclusively in a specific group of bacteria and remain structurally conserved despite extreme sequence divergence. The fold forms a homodimer with a conserved interface, suggesting shared oligomerization state across all members. Biophysical analyses reveal reversible unfolding with chemical denaturants and computational studies suggest a self-tying folding pathway via a slipknot intermediate (monomer and dimer). These findings provide a model system to explore the evolutionary, structural, and functional implications of knotting and its role in shaping protein architecture.

Deciphering the link between protein sequence and structure remains a fundamental challenge in structural biology (1). While proteins exhibit vast sequence diversity, they often retain conserved structural motifs. Among the least understood are knotted topologies—where the polypeptide chain forms an entangled structure (2–6). A protein knot is a topological feature where the protein chain forms a real knot when its ends are joined. If you pull on the ends, the chain tightens into a knot similar to those seen in everyday life. These knots are often embedded in the active sites of proteins or form transport channels in transmembrane proteins (7), yet their relevance to the enzymatic or biological functions conducted (6, 8, 9), as well as their evolutionary origins and implications, remains largely unexplored (10, 11).

A knot is a weave, tying a string around itself. Their complexity is defined by the number of crossings after complete simplification without cutting the chain. Based on the KnotProt database (12), six types of knots have been found in proteins. The most typical is the simplest, a $3_{1}$ knot (13–15). More complex knots are $4_{1}$, $5_{2}$, $6_{1}$ (16), $7_{1}$ (17), and double knot $3_{1}$#$3_{1}$ naturally formed by proteins (18, 19) or tandem repeat knotted motif (20, 21). Thus, the number of knot types observed in proteins is small compared to those in mathematics and those observed in polymers.

Topological analysis of high-confidence AlphaFold models, with results in AlphaKnot Database (22), has revealed that knotted proteins constitute roughly 0.4% of all proteins in any given proteome, with the majority adopting a simple trefoil knot and occurring across bacteria, archaea, and eukaryota without strong taxonomic bias. Notably, no knotless bacterial proteome has been identified, and four highly conserved knotted protein families—including SAM synthases and several SPOUT-family methyltransferases—are present in the vast majority of proteomes (23).

Based on experimentally confirmed structures, knotted motifs are present in mitochondrial, transmembrane, and globular proteins; they are found in at least 30 families (7) and represent 12 folds, each with a unique geometrical motif (24). Thus knotted fold is rare compared to all known folds but it is strictly conserved in 3D structure even between proteins with extremely low sequence similarity within the same family (25). On the other hand, compared to experimental structural databases, analysis shows that additional folds and topologies can be identified (23, 26, 27) in the structures predicted by AlphaFold and deposited in AlphaKnot (28). One such unclassified and very well conserved motif with no experimentally determined single structure is shown here to adopt a knotted fold within the well-established β-solenoid family, a structural class that has so far comprised exclusively unknotted members.

Solenoid proteins are defined by repeating structural motifs forming elongated, helical architectures. These folds, found in at least 14% of all proteins (29), consist of tandem repeats that may include one or several segments of secondary structure such as α-helices, β-strands, and 3_10_-helices. A prominent subgroup, the β-solenoids (30, 31), represents a distinct and functionally diverse class, characterized by their elongated, regular structures built entirely from repeating β-strands stacked into solenoid-like arrangements that wind around a central axis. The number of “sides” of the solenoid dictates the cross-sectional geometry, which can be triangular, quadrilateral, or more complex (32). The modularity and variability of these repeats not only underpin diverse biological functions (33, 34) from molecular recognition to involvement in amyloid formation but also highlight the structural diversity of solenoid folds (35). These various biological functions make solenoids attractive targets for protein design with great success (36).

The β-solenoid proteins are well represented and classified in the CATH database (37). Right-handed β-helices, often associated with glycan binding, are classified under CATH code 2.160.20, with pectate lyases as examples (38). Left-handed β-helices, are found under CATH code 2.160.10, and include spruce budworm antifreeze proteins (39).

Herein, we present a novel Knotted Solenoid fold and identify key differences establishing them as a distinct protein fold within the broader β-solenoid group. Most notably, we observed an order-shifted coil arrangement, with the fourth coil positioned between the second and third—a shift we term “skip-and-backtrack.” The resulting loop allows chain threading during folding, enabling formation of a 3_1_ knot, a feature not observed in any other experimentally solved β-solenoid protein. Building upon this finding, we present evidence of novel knotted topology within a specific solenoid fold previously thought to be unknotted.

We expressed five representative proteins of this novel Knotted Solenoid fold, and solved crystal structures via X-ray crystallography for three of them. Based on X-ray crystallography, SEC-MALS, and bioinformatic analysis, we propose that all members of this group form homodimers. The thermal stability of selected representatives was evaluated using differential scanning calorimetry (DSC) and differential scanning fluorimetry (DSF). Far-UV circular dichroism (CD) was used to monitor unfolding by chemical denaturation. Furthermore, we performed extensive theoretical studies of the functional and taxonomical annotations, as well as compared Knotted Solenoids to other solenoid folds, and analyzed their free-energy landscape based on a structure-based model.

## Results

A thorough analysis of the knotted models in the AlphaKnot database (22) led to a finding of a distinct knotted fold resembling β-solenoid proteins, which we name Knotted Solenoid. Based on a single “reference” protein, we found the fold to be conserved in 184 proteins by searching for structurally and sequentially similar proteins (FoldSeek (40), HMMER (41), see *Materials and Methods* and *SI Appendix*, Fig. S1 for details). We assessed the crystallizability of all 184 identified Knotted Solenoid proteins using XtalPred (42) and CRYSTALP2 (43) and selected five with the highest probability (Table 1). We also identified an additional 303 unique proteins with the Knotted Solenoid Fold in the NCBI Protein database, identified based on sequence similarity with high alignment coverage and low E-values compared to the reference group. The topology of those structures predicted by the AlphaFold model were verified to correspond to 3_1_.

### Crystal Structures Reveal a Novel 3_1_ Knotted Fold.

**Fig. 1.**
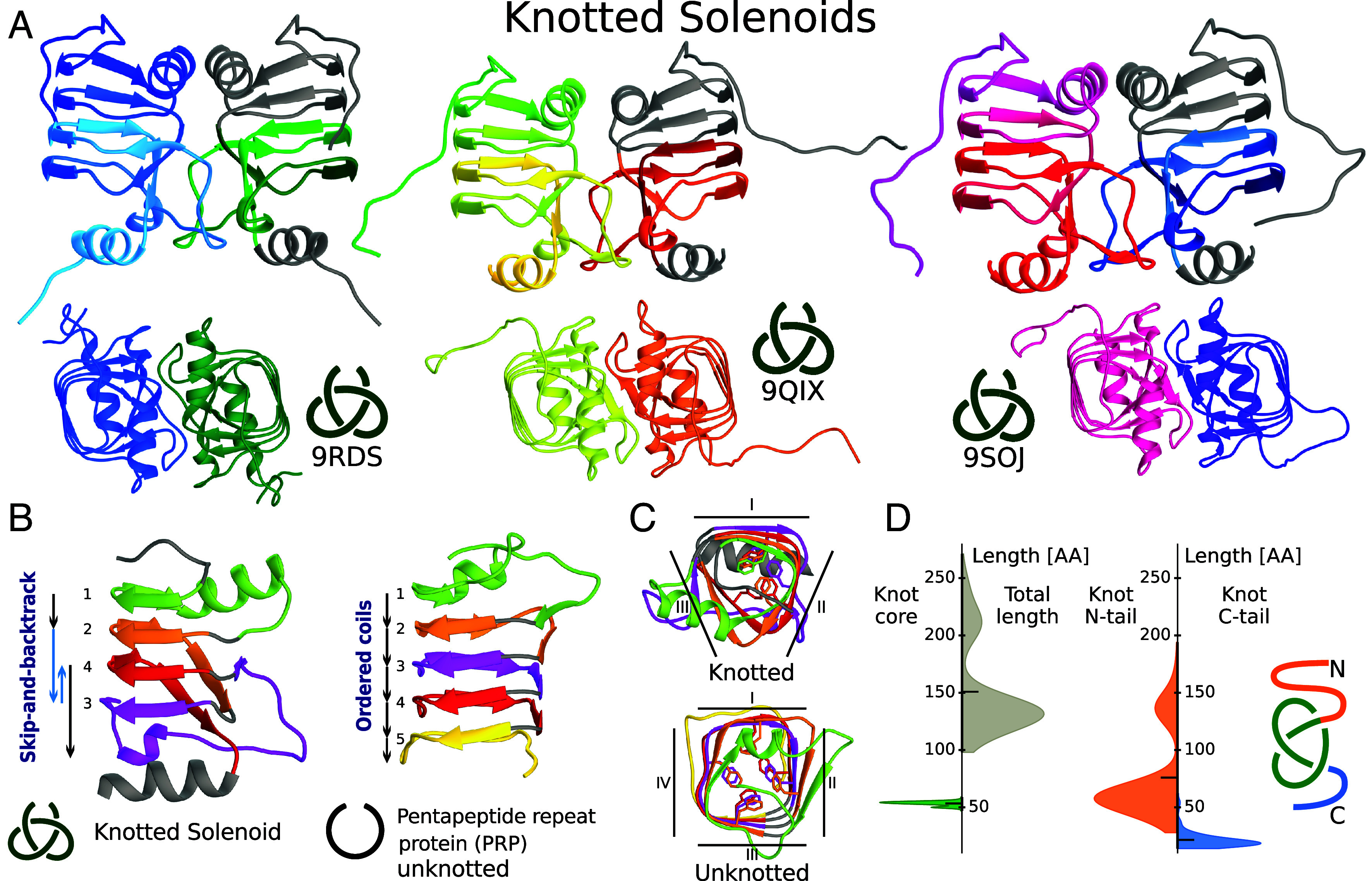
Crystal structures of Knotted Solenoid proteins with PDB ID: 9RDS, 9QIX, and 9SOJ. (*A*) Cartoon representations of structures viewed from the side and top. For each dimer shown from the side, chains on the *Left* are colored by gradient from N to C terminal (blue to cyan, green to yellow, pink to red), while the chains on the *Right* highlight the knot core. (*B*) Comparison of Knotted Solenoid (9RDS, residues 13–127) with its closest unknotted homolog, a pentapeptide repeat protein (PRP) (UniProtKB: A0A4R2TS65, residues 56–173). Coils are colored and numbered to show order; in Knotted Solenoids a disrupted “skip-and-backtrack” order is observed, absent in unknotted solenoids. (*C*) *Top* view highlights other features. Knotted Solenoid coils consist of three parallel β-sheets connected by short turns, compared to four in PRPs. In both, phenylalanines form a repeating inward-pointing pattern. (*D*) Violin plots of knot length statistics, with longer marker showing mean values.

We expressed and purified five proteins (Table 1) and solved three crystal structures from *Brevundimonas* bacteria with UniProtKB IDs A0A653LYW1 (PDB ID: 9RDS), A0A2W6Z6R8 (PDB ID: 9QIX), and A0A1V1V225 (PDB ID: 9SOJ) (Fig. 1), at 1.22 Å, 1.66 Å and 1.90 Å resolution, respectively (see *SI Appendix*, Table S1 for diffraction data collection and refinement statistics). The crystal asymmetric units are composed of a single chain (PDB ID: 9RDS) and two chains forming a homodimer (PDB ID: 9QIX and PDB ID: 9SOJ; Fig. 1).

**Table 1.** Summary of purified representative proteins with the novel Knotted Solenoid fold and their abbreviations

UniProtKB ID	Abbrev.	Organism	MW [Da]
A0A653LYW1	9RDS	*Brevundimonas sp.* G8	14,192
A0A2W6Z6R8	9QIX	*Brevundimonas sp.*	14,112
A0A1V1V225	9SOJ	*Brevundimonas sp.* SH203	14,052
A0A0Q7SN77	γ	*Brevundimonas sp.* Root1423	13,694
A0A4P8QH87	ϵ	*Brevundimonas sp.* SGAir0440	13,994

The proteins with PDB ID: 9RDS, 9QIX, and 9SOJ share 79% sequence identity (E-value: 7 × 10^−80^). Their structural superposition indicates high-level similarity, with a rmsd (RMSD) of 0.61 Å across 115 C_*α*_ atom pairs, and 0.46 Å across 122 C_*α*_ atom pairs (9RDS-9SOJ).

Using methods for topology analysis on open curves (44), we can say that these structures have a well-defined right-handed +3_1_ knot in all crystal structures. In these proteins, the knot core spans residues 55 to 116 (9RDS and 9QIX) or 62 to 121 (9SOJ), comprising nearly half of the structure. This knot is effectively stabilized by long tails: 55 residues from the N-terminal, 16 (PDB ID: 9RDS), 12 (PDB ID: 9QIX), and 11 (PDB ID: 9SOJ) residues from the C-terminal, thus is deep, making spontaneous unknotting highly unlikely.

The analysis of all 184 Knotted Solenoids shows the knot motif is compact regardless of protein length. The knot core comprises approximately 50 residues, while full-length proteins are typically 100–160 residues, reaching 250 if additional N-terminal domain is present. The knot is classified as deep, with both termini longer than 10 residues. C-terminal tails range from 14 residues with 22 residues on average, while N-terminal tails span 28–100 residues without the extra domain and up to 200 with it (Fig. 1*D*).

The structures of the Knotted Solenoid fold match the features of the broader β-solenoid group (32), forming a parallel coiled formation. Each coil consists of three β-strands connected with short β-arcs (31). The average coil axial rise distance between C_*α*_ atoms (residues 21 to 26 and their parallel positions) for proteins with PDB IDs: 9RDS, 9QIX, and 9SOJ is 4.8 Å with a SD of 0.1 Å, allowing the formation of intercoil hydrogen bonds and is also within the expected value range. A characteristic well-defined hydrophobic core (Fig. 3) is also observed. However, in the case of Knotted Solenoids, some hydrophobic residues’ sidechains are also oriented toward the surface, forming a hydrophobic interface, which in turn results in a dimeric structure as observed for the proteins with PDB IDs 9RDS, 9QIX, 9SOJ, each of which forms a dimeric assembly in the crystal, whether within the asymmetric unit or through crystallographic symmetry (*SI Appendix*, Fig. S8).

### Knotted Solenoid Adopt Dimeric Structures.

**Fig. 2.**
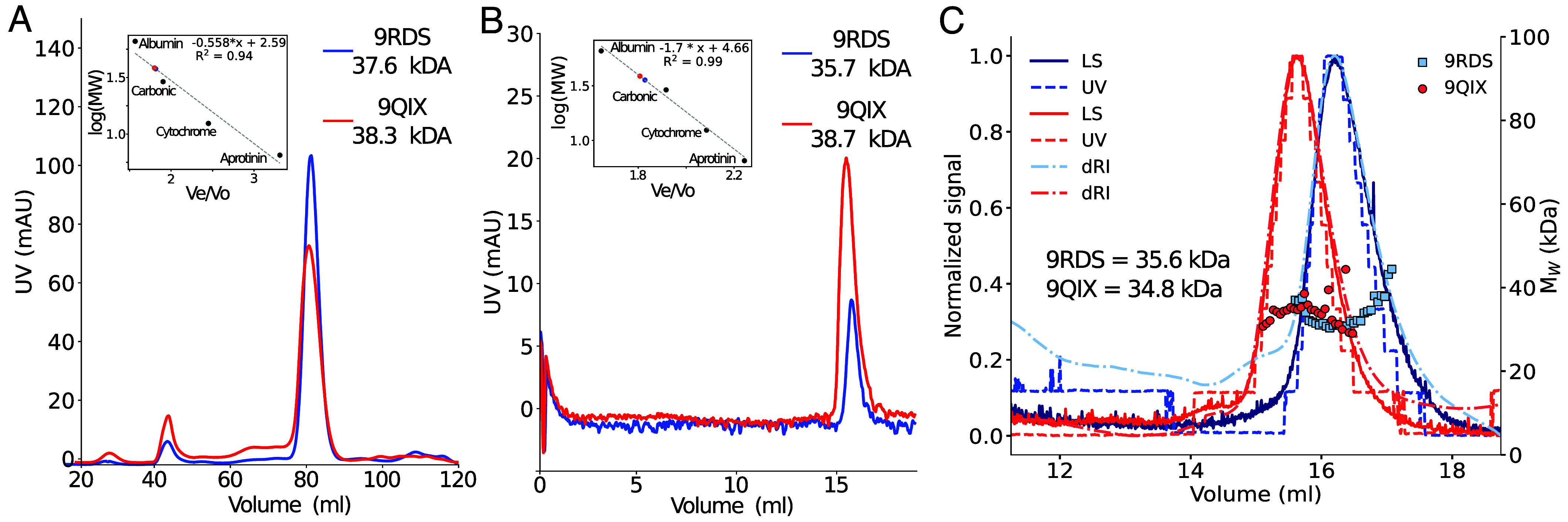
Characterization of the size and compactness of Knotted Solenoid with PDB ID: 9RDS and 9QIX under native conditions. Elution and oligomerization analysis of Knotted Solenoids 9RDS (blue) and 9QIX (red) in the native state. (*A*) FPLC purification profiles; *Inset* shows calibration curve for MW estimation. (*B*) Analytical SEC profiles; *Inset* shows standard curve for MW estimation from elution volume. (*C*) SEC-MALS analysis showing light scattering (LS), ultraviolet absorbance (UV), and differential refractive index (dRI) signals, normalized to 0 to 1 by dividing each spectrum by its maximum across the elution volume. Analysis for protein γ in *SI Appendix*, Fig. S14. Protein samples in SEC buffer (50 mM Tris pH 7.4 at 25 ^°^C, 200 mM NaCl, and 5% glycerol).

In Knotted Solenoids, unlike unknotted ones, some hydrophobic residues are exposed on the surface of a monomer, forming a hydrophobic interface that promotes dimerization. The oligomerization state was first assessed during protein purification using a size-exclusion column, with fast protein liquid chromatography (FPLC) elution profiles shown in Fig. 2*A* for proteins 9RDS (blue) and 9QIX (red). A calibration curve was used to estimate molecular weights (MW) based on elution volumes. The linear fit shows a strong correlation, supporting the estimated MW values: 37.6 kDa for protein with PDB ID: 9RDS and 38.3 kDa for 9QIX. These are notably higher than their theoretical molecular weights of 14.2 kDa and 14.1 kDa, respectively. The oligomeric state was also determined on the purified proteins using a different size-exclusion column, and the profiles are shown in Fig. 2*B*. The results for the MWs of Knotted Solenoid with PDB ID: 9RDS and 9QIX were 35.7 kDa and 38.7 kDa, respectively, and in agreement with the values determined during purification, Fig. 2*A*.

Fig. 2*C* shows SEC-MALS data supporting a dimeric state for both proteins. The MWs of Knotted Solenoid 9RDS and 9QIX are 35.6 and 34.8 kDa, respectively, roughly matching twice their theoretical weight. Single, well-defined peaks in Fig. 2*C* suggests that the dimeric form is stable and likely the predominant oligomeric state under the experimental conditions. Analysis for the γ protein for which we did not obtain a crystal structure also suggests a dimer (*SI Appendix*, Fig. S14).

**Fig. 3.**
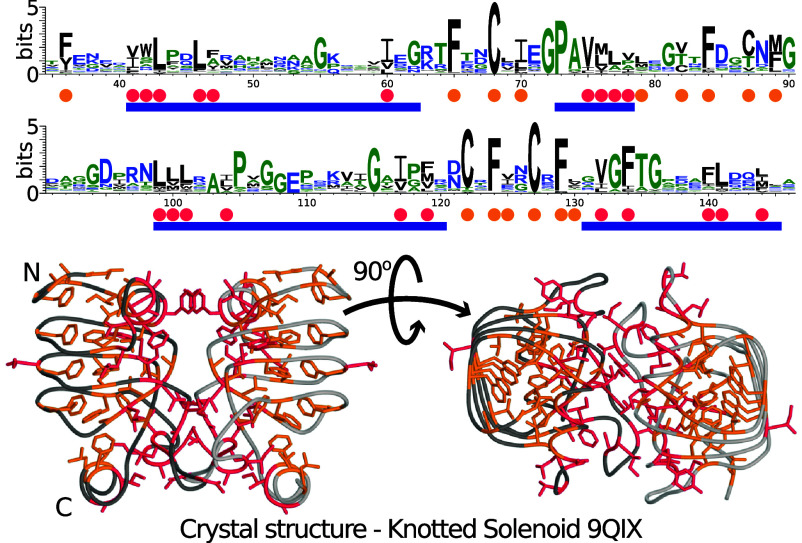
Knotted Solenoid dimer interface and hydrophobicity. The analysis is based on the protein with PDB ID: 9QIX crystal structure (trimmed terminal six residues in both chains). The *Upper* panel shows the sequence logo for the Knotted Solenoid group with residues colored by hydrophobicity. Below the sequence logo, the blue line represents sections of the protein forming an interface. Orange dots above represent especially conserved hydrophobic residues with side chains turned toward the channel, while pink ones represent those turned outward. The *Lower* panel shows side and top-down line representations of the dimer structure. Hydrophobic residues are colored according to the orientation of side chains: Orange indicates side chains turned toward the channel, while pink marks those turned outward.

To assess whether dimerization occurs in every representative of the Knotted Solenoid group, we performed a sequence alignment of all the members and analyzed the sequence logo (Fig. 3). Results show that hydrophobic residues are primarily located on one side of the protein involved in forming an interface, although the specific residue type and its position are not strictly preserved in the Knotted Solenoid group. Conserved hydrophobic residues outside this region have their side chains turned inward toward the Knotted Solenoid channel. Specific residues are also much more conserved and with strictly defined positions, most likely to keep the Knotted Solenoid structure stable (Fig. 3). Consistently, AlphaFold Multimer predicted dimeric structures matching the crystallographic interface also for structures without the crystal structure solved. Overall, we conclude that all members of the Knotted Solenoid group should form dimeric structures.

### Knotted Solenoid Proteins Exhibit a Disrupted Loop Order.

The Knotted Solenoid fold begins with a flexible N-terminus, followed by the first coil in the sequential order (Fig. 1*B*, green color). This coil consists of two β-strands aligned with the helical structure, followed by an outward-facing α-helix, forming the first protrusion. The second coil in the sequential order (orange) consists solely of β-strands. It skips one rotation with the third coil (pink) then backtracking via a long (around 25 residues) extended linker, creating the second protrusion, extending in the same direction as the first. The fourth coil in the sequential order, similarly to the second, skips a rotation and connects to the C-terminal α-helix, which caps the channel. The overall structure remains well-aligned. This observation is further verified on the Knotted Solenoid coordinate profile (45) where the vertical shift between the second and the third loop is over twice as big with the third coil then shifting backward (Fig. 1).

The “skip-and-backtrack” order makes the Knotted Solenoid fold stand out from the other β-solenoid groups and is possible via an extended, backtracking linker, long enough to allow for the threading of the third loop resulting in the formation of a 3_1_ knot via an intermediate slipknot stage during folding (Fig. 6; see the section about folding). In other words, the knotted topology emerges as a direct consequence of the “skip-and-backtrack” shift during folding which is determined in the sequence. This knot is stabilized in two ways: via hydrogen bonds forming between the loops, and with the C-terminal α-helix, greatly reducing the probability of incidental unfolding.

The stability of both the knot and the fold is also evident from all-atom explicit-solvent molecular dynamics simulations and from a generative deep learning model, BioEmu (46). In four independent 200-ns trajectories performed for the Knotted Solenoid proteins (monomer and dimer), the overall conformations of the proteins remain largely unchanged (*SI Appendix*, Fig. S18). To further enhance the sampling of the conformational space, we employed the BioEmu method. In both approaches to conformational sampling (the RMSF values [Å] of each residue), apart from terminal fluctuations, only some fluctuations in loops were observed (skip-and-backtrack loop between the third and fourth coils, which are part of the knotted core of the proteins).

### Nearest Unknotted β-Solenoid Proteins Are from the Pentapeptide Repeat Family.

To investigate how Knotted Solenoids differ from unknotted proteins, we analyzed proteins similar to 9RDS. We identified 21 unknotted pentapeptide repeat proteins [PRP; CATH ID: 2.160.20.80, over 100,000 representatives in Gene3D (47)]. Their predicted structures are unknotted, two-domain proteins: a β-helix N-terminal domain and a C-terminal domain of α-helices. Sequentially, their solenoid domain aligns with 3_1_ Knotted Solenoids, despite structural differences (*SI Appendix*, Fig. S2), and they share conserved residues, mainly Phe, Cys, and Gly.

**Fig. 4.**
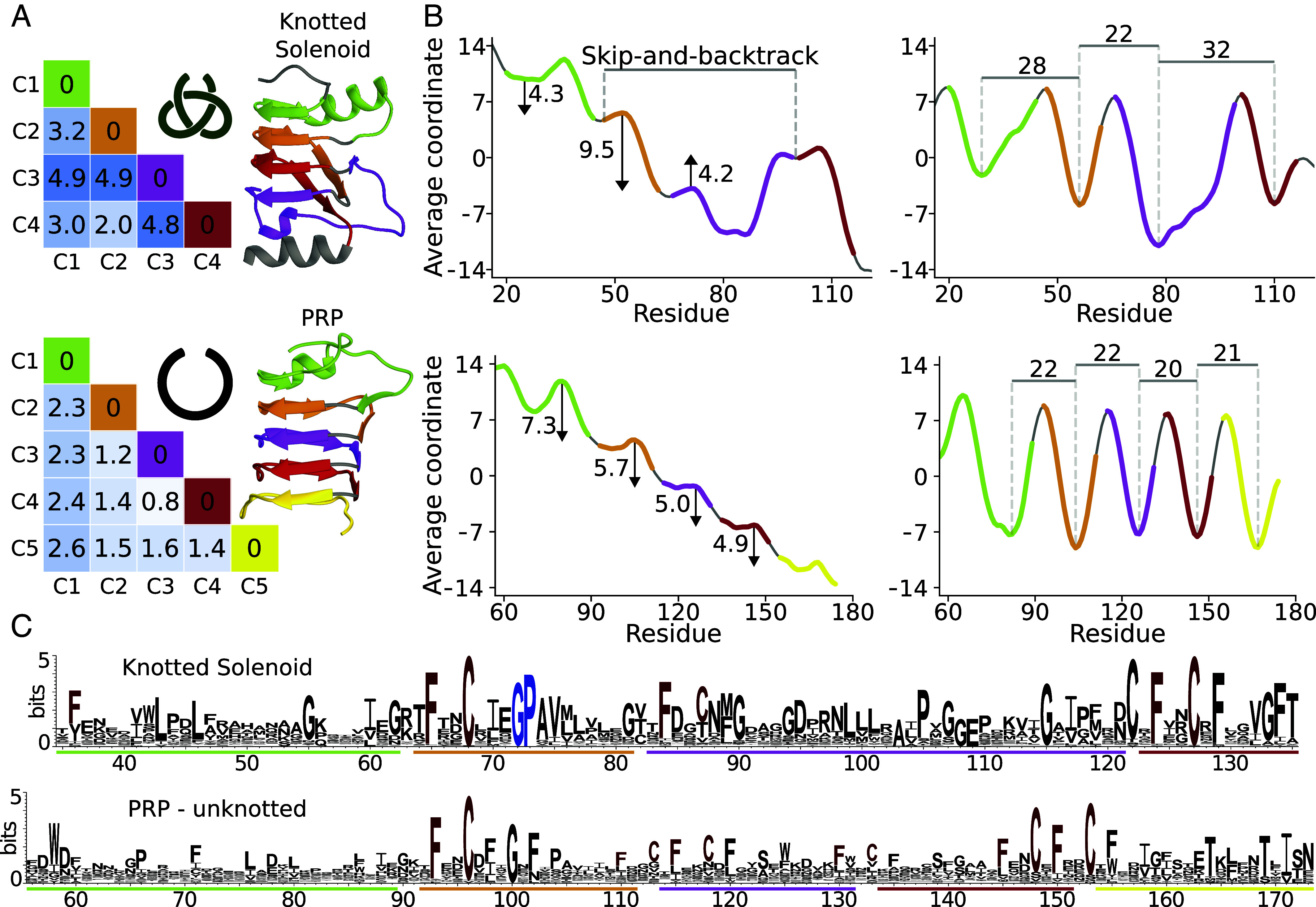
Characterization of the Knotted Solenoid with comparison to pentapeptide repeat protein. The analysis is based on the protein with PDB ID: 9RDS (trimmed to residue range 13 to 127) and unknotted PRP (UniProtKB ID: A0A4R2TS65, trimmed to range 56 to 173) with all panels using the same color coding for each coil (Labeled C1 to C4 or C5 in the sequence order). (*A*) Matrices visualize the RMSD similarity of individual coils. (*B*) Two sets of coordinate profiles are shown for the y and z axis respectively. A clear difference can be observed between the two proteins, with Knotted Solenoid presenting a “skip-and-backtrack” shift not present in the PRP. (*C*) Fragment of the sequence logo for proteins with Knotted Solenoid fold without additional domain and PRP. Especially conserved residues repeating at the beginning of each coil are marked with red color, and a glycine-proline at the beginning of the knot core is marked with blue.

Fig. 4 highlights the structural distinction. Unknotted PRPs have a well-ordered quadrilateral β-helix of four parallel β-sheets with repeating Phe residues. Knotted Solenoids form a triangular helix with fewer Phe, and only two sides maintain the stacking order; the third side is disrupted by the knot.

The difference between the two types of β-solenoids is especially clear when looking at the coordinate profiles (Fig. 4*B*). The *y*-axis coordinate profile reveals a distinct “skip-and-backtrack” shift in Knotted Solenoid, not observed in the PRP. Because of that, the *z*-axis coordinate profile also appears to be less regular in the nontrivial protein, with longer coils required to perform the shift.

The identification of an unknotted β-solenoid from the “pentapeptide repeat” family (UniProtKB ID: A0A4R2TS65) as the closest relative to our representative Knotted Solenoid with PDB ID: 9RDS provides a second instance of topological variation within a shared fold, complementing the well-known aspartate/ornithine carbamoyltransferase (ATC/OTC) protein family (InterPro) (48, 49). Notably, unlike ATC and OTC, which belong to the same protein family, the pentapeptide repeat protein and Knotted Solenoids originate from distinct families. They also differ in sequence identity: the solenoid pair shares only 14%, a contrast to identity as high as 38% in ATC/OTC proteins (11). This makes the solenoid pair particularly advantageous for dissecting the impact of topology on distantly related proteins. They open paths for comparative analyses of topological effects across varying degrees of sequence conservation.

### Knotted Solenoid Fold Represents a Distinct Fold.

We investigated whether Knotted Solenoids should be classified within the existing β-solenoid group in the CATH database or as a separate category. Among other experimentally determined structures with the β-solenoid fold in the CATH database (37), the structures with the highest local structural similarity are structures with PDB IDs: 2QYU and 4PMH. The first structure belongs to the “E3 ubiquitin-protein ligase SopA” group (CATH ID: 20.160.20.80), while the second structure belongs to the “single-stranded right-handed β-helix, pectin lyase-like” (CATH ID: 2.160.20.10). This has been further verified by computing a structure-based tree where structures from the aforementioned two groups are consistently located near Knotted Solenoids (*SI Appendix*, Fig. S4). None of these structures exhibit the “skip-and-backtrack” shift or the resulting knotted topology characteristic of the Knotted Solenoids.

To further verify that Knotted Solenoids represent a novel protein fold, we used the TED (The Encyclopedia of Domains) database (50), which enables the detection of folds in AlphaFold-predicted structures and attempts classification within the CATH system. No known CATH domain was assigned to the Knotted Solenoid proteins. Furthermore, we found exactly one match in TED dataset of novel folds via structural comparison, which corresponded to one of the 184 Knotted Solenoid proteins analyzed in our study.

We also tested a scenario in which Knotted Solenoids lack the “skip-and-backtrack” shift while the remainder of the sequence is left unchanged, to assess whether potential unknotted ancestors of Knotted Solenoids could be detected. However, this approach identified only proteins belonging to the Knotted Solenoid group, further supporting the view that Knotted Solenoids possess a distinct sequence (*SI Appendix*, Fig. S5).

We have also investigated all experimental structures used as templates by the AlphaFold model to predict the Knotted Solenoid fold (*SI Appendix*, Fig. S1). Fifty different folds (based on CATH classification) were used with 12 of them in at least 10 Knotted Solenoid structures. These include other β-solenoid folds, most commonly single-stranded right-handed β-helix, pectin lyase-like (2.160.20.10), and E3 ubiquitin-protein ligase SopA (2.160.20.80). However, other classes have also been found: “Mainly Alpha” fold—cytochrome c-like domain (1.10.760.10) and “Alpha Beta” fold: glycosyl transferase family (3.90.1480.20), likely used in structures with an additional domain at the N-terminus of the Knotted Solenoid proteins. None of those template structures have a knot. Comparison (measured with RMSD) between the crystal structures and the structures predicted by the AlphaFold model is around 0.53 Å across 116 pairs for protein with PDB ID: 9RDS, 0.57 Å across 119 pairs for protein with PDB ID: 9QIX and 0.64 Å across 122 C_*α*_ pairs for protein with PDB ID: 9SOJ, with the differences mainly at the flexible ends of the structures. This shows that AlphaFold was able to model a fold not observed in the template structures, regardless of their topology *SI Appendix*, Fig. S9).

Finally, we analyzed “Biological Assemblies” from the PDB database for all β-solenoid proteins and identified a set of features distinguishing Knotted Solenoids from other members of the 2.160 CATH lineage. We did not find any unknotted β-solenoid biological assembly that forms a symmetric dimer through a hydrophobic interface, with triangular channels arranged side-by-side and the chains oriented in parallel (N-to-C) (*SI Appendix*, Fig. S6).

Taken together, these results show that the Knotted Solenoid represents a distinct fold within the established CATH lineage of β-solenoids. The fold itself is also identified in the TED database as a novel fold. Its unique features include a nontrivial topology arising from a distinctive “skip-and-backtrack” shift. This shift causes exposure of the hydrophobic side chains to the surface of the native monomer and outside of the triangular solenoid channel, and as a result, formation of a dimer. This is also paired with the fact that in no other β-solenoid did we find any symmetric side-by-side dimers with parallel (N-to-C) chains.

### Novel Knotted Solenoid Fold Is Highly Conserved in the Brevundimonas Bacteria.

Sequential clustering at 30% identity threshold returned 27 representative proteins. Notably, the three largest clusters account for over 50% of all sequences, with five experimentally purified proteins found within two of these clusters. The lowest sequence identity (6.1%) was observed between proteins A0A1L3ZWT6 and A0A3Q9UYF8. The identity relationships among the 27 representative proteins are visualized in *SI Appendix*, Fig. S17.

The sequence logo for all Knotted Solenoid proteins highlights a highly conserved phenylalanine, followed by a cysteine three residues later (Fig. 4*D*; full logo *SI Appendix*, Fig. S3). These residues consistently appear at the start of the second, third, and fourth coils. When aligned, they form a vertical pattern, reflecting the β-solenoid’s helical structure. Moreover, a highly conserved glycine-proline is observed at the beginning of the knot core, which, due to relatively short side chains, might be important for the correct folding process.

Despite the conservation of cysteine pairs in the sequence and their close spatial proximity, we did not observe disulfide bonds in any of the structures. The refined sidechain orientations place the sulfur atoms at distances and geometries that are not compatible with disulfide formation, making such a linkage unlikely. Such an observation is not uncommon in β-solenoid folds (32).

Taxonomic analysis shows that all Knotted Solenoid proteins originate from bacteria, with 51% belonging to the *Caulobacteraceae* family, particularly the *Brevundimonas* genus (43%). Family *Caulobacteraceae* comprises Gram-negative, often stalked bacteria reproducing by budding, with environmental prevalence in soil and aquatic habitats. They have also been implicated in human infections and are occasionally linked to hospital-acquired infections (51). Functional analysis using DeepFRI (52) found no hits exceeding the standard confidence threshold for either structural or sequence-based evaluation of function.

### Stability Against Thermal Denaturation.

**Table 2.** Apparent stability data obtained by analysis of the DSC (protein concentration 50 μM, ΔH and $T_{\mathrm{ma}}$ include fitting errors when applicable), and DSF spectra (concentration 11 μM)

Protein	$\Delta H_{\mathrm{app}}$ [kJ/mol]	$T_{\mathrm{ma}}$ [^°^C]	$T_{\mathrm{ma}}$ [^°^C]
	DSC	DSC	DSF
9RDS	77.3 ± 20	54.6 ± 0.2	51.5 ± 0.9
9QIX	64.4 ± 6	60.4 ± 0	58.7 ± 0.2
9SOJ			56.7 ± 0.4

Moreover, for protein γ and ϵ, we report $T_{\mathrm{ma}}$ from DSC experiment as 67.7 and 57.8 ^°^C respectively. In both experiments, the proteins were in the SEC buffer (50 mM Tris pH 7.4 to 5.6 at 25 to 90 ^°^C, 200 mM NaCl, and 5% glycerol).

**Fig. 5.**
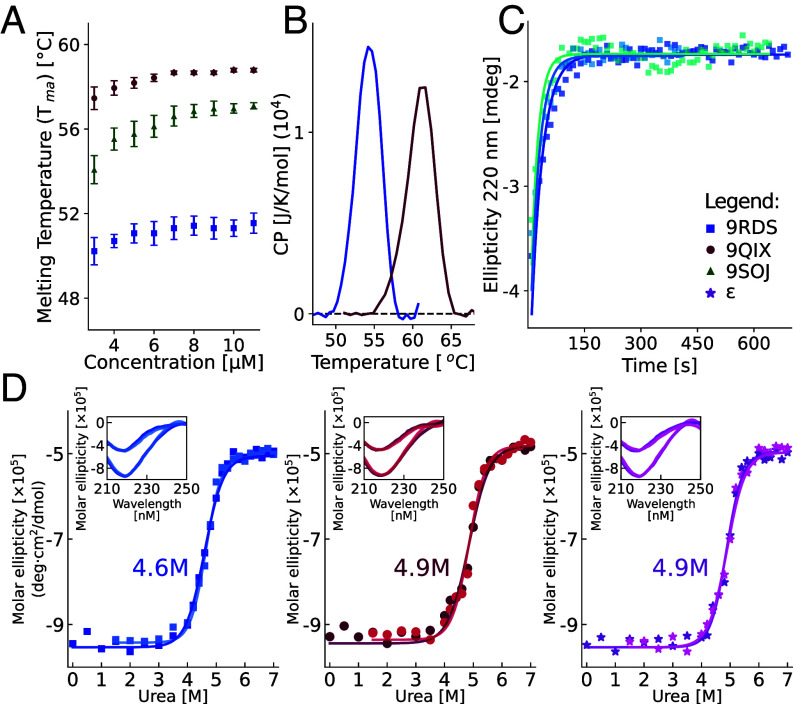
Stability of Knotted Solenoid proteins against thermal and chemical denaturation. (*A*) Apparent melting temperatures of 9RDS, 9QIX, and 9SOJ from DSF (SYPRO Orange) at increasing protein concentrations, each measured in triplicate. (*B*) The heat capacity signal as a function of temperature. The maximum represents the midpoint of thermal unfolding while the area under the curve is an estimate of the $\Delta H_{\mathrm{app}}$, see Table 2. (*C*) Time course of unfolding in 4 M guanidinium chloride for protein 9RDS, measured via far-UV CD, monitored at 220 nm; three independent replicates shown. The unfolding half-life of the protein is 26 ± 6 s. Fitting parameters can be found in *SI Appendix*, Table S3. (*D*) Fitting of molar ellipticity at 220 nm from far-UV CD spectra at varying urea concentrations for proteins 9RDS, 9QIX, and ϵ. Darker lines indicate unfolding and lighter ones refolding. *Insets*show boundary spectra: unfolded proteins in 7 M urea exhibited reduced signal, while refolded samples in 1.5 M urea closely matched native spectra in 0 M urea. Parameters of two-state model used for fitting can be found in *SI Appendix*, Table S2. In all experiments, the proteins were in the SEC buffer (50 mM Tris pH 7.4 at 25 ^°^C, 200 mM NaCl, and 5% glycerol; CD with no glycerol).

Thermal stability analysis using differential scanning fluorimetry (DSF) revealed a range of apparent melting temperatures ($T_{\mathrm{ma}}$), from 51 to 59 ^°^C (SD not exceeding 0.9 ^°^C), comparable to other β-solenoid proteins (53, 54). Additionally, DSF performed at increasing protein concentrations (2 to 11 μM), revealed a slight increase of the $T_{\mathrm{ma}}$ with higher protein concentrations, reaching a plateau at around 5 μM (Fig. 5*A*). As the unfolding transitions appear irreversible—evidenced by the sharp signal loss after the melting peak (*SI Appendix*, Fig. S12)—the reported $T_{\mathrm{ma}}$ values reflect apparent, not true, melting temperatures. To confirm this, we performed a heating–cooling–reheating experiment (*SI Appendix*, Fig. S13). During cooling, fluorescence rapidly increased. Reheating caused a gradual signal loss without additional inflections, confirming irreversible denaturation.

DSC experiments shown in Fig. 5*B* were used to estimate the $T_{\mathrm{ma}}$ and $\Delta H_{\mathrm{app}}$, parameters shown in Table 2. In this case, thermal denaturation was again not reversible, and a sharp drop in heat capacity ($C_{P}$) at high temperatures likely indicates protein aggregation (*SI Appendix*, Fig. S15). As a result, the calculated apparent melting temperature ($T_{\mathrm{ma}}$) and enthalpy of unfolding ($\Delta H_{\mathrm{app}}$) should be considered as estimates, representing the lower bound of $T_{\mathrm{ma}}$ and the upper bound of ΔH.

### Knotted Solenoid Proteins Unfold Reversibly in Urea.

Unfolding was also assessed by far-UV CD spectroscopy in the presence of urea, revealing a simple two-state unfolding model. The transition midpoints for 50 μM samples were calculated to be 4.62 ± 0.02 M for protein 9RDS, 4.89 ± 0.03 M for 9QIX, and 4.91 ± 0.02 M for ϵ. Refolding experiments confirmed full reversibility with no hysteresis (Fig. 5*D*). For protein 9RDS, we also examined the effect of protein concentration on stability. Upon a tenfold dilution to 5 μM, the transition midpoint shifted slightly to 4.3 ± 0.07 M as anticipated for a dimeric system (*SI Appendix*, Fig. S11).

Given the residual signal observed at 7 M urea, we extended the unfolding experiments to more extreme conditions. We started by increasing the urea concentration up to 9 M, and a residual far-UV signal was still observed. A similar observation was made with 8 M guanidinium chloride.

Studies of the unfolding kinetics of solenoid 9RDS in 4 M guanidinium chloride reveal one phase with half-life values around 26 ± 6 s. In the case of solenoid 9QIX the unfolding process is so fast that within the dead time of manual mixing (around 18 s), we can already see the unfolded protein (Fig. 5*C*; additional longer measurement in *SI Appendix*, Fig. S16). Based on the available literature (55), it appears that knotted proteins unfold much more slowly than those with trivial topology (half-life values below 6 s), but the difference could also result from differences in primary sequences.

### Knotted Solenoid Can Self-Tie via a Slipknot Topology.

**Fig. 6.**
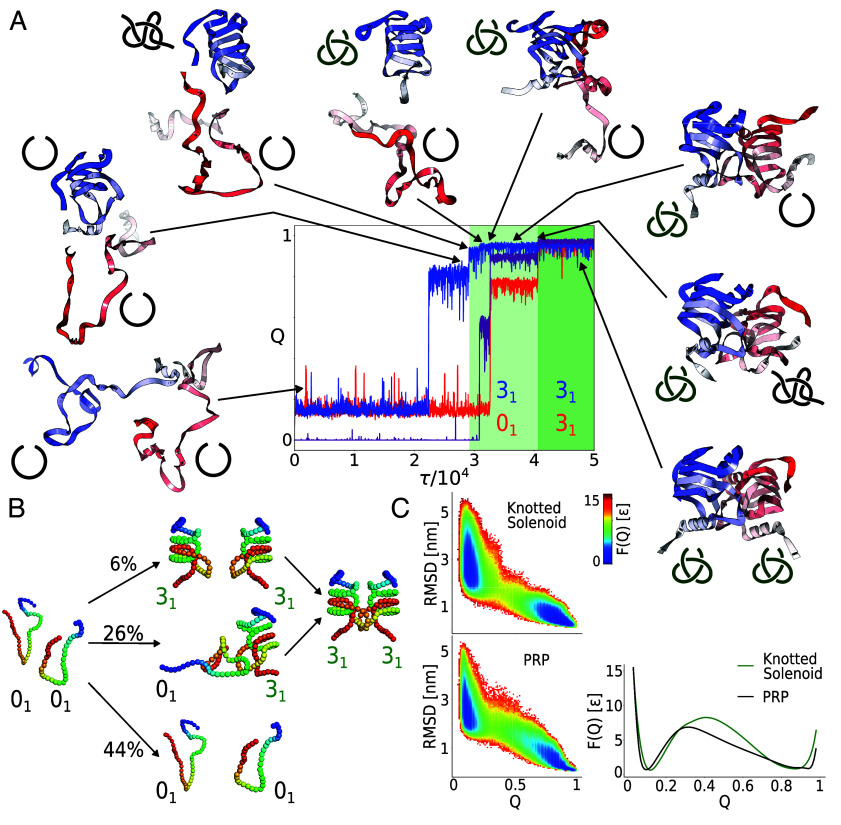
Folding mechanism of the Knotted Solenoid dimer (PDB ID: 9RDS) based on a structure-based coarse-grained model. (*A*) Example of a folding trajectory shown based on the native contacts (Q) as a function of time. Each unit of the dimer is shown in red and blue, and the contacts at the interface are shown in magenta. Light and dark green indicate the formation of the knots. Conformations surrounding the chart are snapshots from the trajectory showing some of the most important steps, including the formation of two knots via slipknot topology. (*B*) Diagram showing folding statistics. In 56% of the simulations, at least one chain folded and knotted successfully. In 26% trajectories, the second chain is folded after forming contacts with the already knotted chain, and in 6% of the trajectories, the second chain folds independently. In the reminding 24% of simulations, the process takes too long to observe either of the aforementioned scenarios. (*C*) Free energy landscape as a function of Q and RMSD for the folding and unfolding of a single chain (independent equilibrium simulation of the monomer) and the structurally closest unknotted solenoid (PRP, shown in Fig. 1*B*) and one-dimensional plot showing F(Q) for both proteins.

To investigate a possible folding mechanism of Knotted Solenoid proteins, we analyzed the results of simulations of protein folding trajectories in both monomer and dimer forms using the example of protein 9RDS obtained from molecular dynamics with structure-based coarse-grained model. In the case of a dimer, the initial state was composed of unfolded and unknotted proteins connected by a very soft spring to the center of mass. Trajectory analysis shows that the native contacts are sufficient for the protein to form a knot by itself (Fig. 6), with the folding efficiency above 40%, a value significantly higher than in the case of other proteins with a $3_{1}$ knot investigated using the same model (7, 56–58) but lower than folding of a designed knotted protein, (PDB ID: 2OUF), from the Yeates group (14). This number, and statistics presented below would be also affected by the longer simulation times of the simulations or effects not taken into account in our model like crowding or encapsulation, which can have significant impact for proteins and polymers with nontrivial topology (59).

From a statistical point of view, in 56% of the trajectories at least one monomer folded successfully. In 26% of the cases, one protein folds (knots) first and then makes contacts at the interface with the second protein chain, followed by the folding of the second protein. On the other hand, the number of cases in which we observed folding of both monomers without any interactions between them prior to dimerization is 6%.

Next, we asked what effect native contacts between proteins have on folding. The proteins form 61 native contacts between themselves, 10 of which are hydrophobic within and outside the knot, *SI Appendix*, Fig. S22. A single monomer folds independently in 2.06 τ. So, we considered only the cases when one monomer folds without forming contacts with the other monomer and the second protein remains in the unfolded state until after a significant fraction, i. e. 50%, of the native contacts in the interface are established. When one monomer folds first and the second remains unfolded until at least 50% of native interface contacts form, the second folds in 1.43 τ (measured from when interface contacts first exceed 50%; see Fig. 6 and *SI Appendix*, Fig. S22). Analysis of the contacts formed between monomers during folding (see histogram in *SI Appendix*, Fig. 22) shows that there are different types of contacts, including hydrophobic ones. Analysis of contacts between monomers during folding shows several types of interactions, including hydrophobic ones. These results suggest that interchain contacts, including hydrophobic ones, accelerate folding.

In nearly all trajectories reaching the folded state, the knot forms by C-terminal threading via a slipknot conformation, whether folding occurs independently or at the hydrophobic interface. Folding of a single monomer, with detailed contact and topology analysis, is shown in *SI Appendix*, Fig. S20.

The process starts with the stabilization of the N-terminal part of the structure in a native-like way (Fig. 6). In this step, we observe the formation of two layers, which involve around 54 residues from the N-terminus. It is followed by the formation of the next two layers in separate events i) by residues 95 to 107, and ii) by residues 60 to 71. In the final stage of folding, we have the 13-residue C-terminal end starting with residue number 109, which threads through the long extended linker region containing residues 72 to 94. Threading is realized by a slipknot arrangement of the C-terminal tail. As a consequence, the protein adopts a $3_{1}$ knot topology. The knotting process takes place exclusively from the C-terminal of the protein as its significantly shorter compared to the N-terminal tail (see histogram; Fig. 1*D*).

The computer simulation results suggest that the protein will fold/tie and unfold/untie on its own. Although we have no evidence to show that from far-UV CD data, the fact that we do not see any hysteresis suggests that these proteins do not require chaperone proteins for tying.

Finally, one may also examine how the presence of a knot influences folding in comparison with an unknotted solenoid, such as the one shown in Fig. 1—the PRP protein, or more precisely, the fragment that forms a β-solenoid (full structure is shown in *SI Appendix*, Fig. S19). In this case, the folding of a single monomer is compared. The free-energy landscape presented as coordinates of Q and rmsd, F(Q, RMSD), indicates an apparent two-state folding process for both knotted and unknotted proteins. In the case of knotted protein, it is a rather more complex process. Herein, the folding funnel appears to be significantly narrower (because the configuration space is spatially restricted by the knot). The difference between the way knotted and unknotted proteins fold is more clearly visible in the Q(τ) presentation (*SI Appendix*, Figs. S20 and S21), where the intermediate state is seen for knotted protein. This shows that F(Q,RMSD) is not an optimal reaction coordinate for this knotted protein. Furthermore, given the sequence differences and the fact that only a fragment of the full-length unknotted protein is simulated, quantitative comparison between the proteins should be interpreted with caution.

All-atom explicit-solvent molecular dynamics simulations and a generative deep learning model approach (BioEmu) for Knotted Solenoids (9RDS, 9QIX, 9SOJ) and unknotted solenoid (PRP protein) show that all folds are stable (will not unfold even locally in 200 ns; and for knotted proteins—also not untie) (*SI Appendix*, Fig. S19). Comparison of the RMSF (Å) value distribution (after structural alignment) shows that in knotted proteins, the two loop regions forming the knot fluctuate more than the rest of the structure in both monomer and dimer, unlike unknotted β-solenoids with regular, uninterrupted structures.

The exact role of the knots in proteins is unclear, and may vary between families. Based on the essentially identical structures of the two protein variants [connected by a flexible linker; PDB ID: 2OUF (14)], studies show that concatenation (knotting) destabilizes (by increasing unfolding rate constants) the protein (60). While in the case of the OTC/ATC family, based on coarse-grained simulations it was suggested that the knot stabilized the structure (61). Fluctuations in the knotted core observed for Knotted Solenoids may indicate that these sites play a functional role, e.g., binding ions or other molecules, as in the case of the knotted SPOUT family (9, 62).

## Summary and Discussion

In this work, we present a Knotted Solenoid fold based on the experimentally determined structures of three proteins. While these proteins share key features with known β-solenoid folds, they exhibit a distinct “skip-and-backtrack” shift, disrupting the continuous coil order and defining them as a novel fold within β-solenoid proteins. This shift, coupled with an extended loop between the third and fourth coils, enables backbone threading during the folding process and the formation of a stable 3_1_ knot, as supported by molecular dynamics simulations (coarse-grained, native-based). The “skip-and-backtrack” shift exposes hydrophobic side chains to the outside, facilitating dimerization. Additionally, crystallography, SEC-MALS measurements, and analysis of conserved residues suggest that all of the Knotted Solenoids assemble into a homodimeric complex.

Based on the low sequence identity (14%) and analyses of scenarios using sequences without “skip-and-backtrack” shift, there is no clear indication that Knotted Solenoids evolved from the unknotted solenoid fold. If such a relationship exists, it could reflect a very early origin that is no longer clearly traceable at the sequence level. Moreover, analysis of Biological Assemblies in the PDB Database did not reveal any unknotted β-solenoid forming a symmetric dimer via a hydrophobic interface with laterally arranged triangular channels and parallel N–C chain orientation.

All this shows that the Knotted Solenoid motif represents a distinct fold within β-solenoid folds. This family presents an opportunity to understand the evolutionary pressures and functional constraints associated with the formation of a knot. Also, a naturally Knotted Solenoid fold and the established purification protocols provide an aid to design future solenoid folds with AI support (36), which is not an easy task, since they are prone to aggregation (35).

Annotations for the Knotted Solenoid proteins within databases are sparse, and their functional role is unknown. However, they appear highly conserved within *Brevundimonas* species, the *Caulobacteraceae* family, which includes environmental Gram-negative bacteria occasionally implicated in human and hospital-acquired infections. While we focused on representatives from the UniProtKB database, we also detected additional candidates in the NCBI Protein and MGnify databases based on sequence similarity and topology consistent with the reference structures, suggesting a wider, yet still limited, distribution of this fold.

Biophysical characterization shows that the knotted fold is stable, but not exceptionally stable (apparent melting temperatures around 55 ^°^C). Chemical unfolding experiments demonstrated full reversibility in urea, with a comparable unfolded signal in high concentrations of guanidinium chloride. Far-UV CD kinetics (in guanidinium chloride) reveal relatively rapid unfolding.

Details of the energy landscape can be deduced from coarse-grained native-based molecular dynamics simulations, which show that proteins can self-tie and untie, and knotting occurs via a slipknot motif at the final stage of folding. The same slipknot mechanism is observed in the case of folding of monomer and after dimerization. Although one protein can fold on its own, hydrophobic contacts formed during dimerization with a folded partner (monomer) accelerates the folding of the second chain.

To conclude, the identification of the Knotted Solenoid fold expands our understanding of protein topology, challenging the conventional view of solenoids as exclusively unknotted structures. This raises questions about the evolutionary and functional significance of entangled solenoid architectures, providing an opportunity for further investigations.

## Materials and Methods

Complete methods with additional details are available in *SI Appendix*.

### Homolog Search and Motif Determination.

After the identification of the first two proteins (UniProtKB ID: A0A7W8HX03 and A0A2X1AF08), FoldSeek (40) was employed on the AlphaFold representative database (afdb50, afdb-proteome, and afdb-swissprot; v4) and PDB to detect structural homologs. Subsequently, a multiple sequence alignment (MSA) was generated using MAFFT (63), which was then used with HMMER (41) to perform sequential searches across the entire AlphaFold database, e-value cutoff: $10^{-3}$. Topology confirmed using topoly package (64).

To identify the common motif sequential and structural alignments were performed. Sequential alignment using the MAFFT package (65) combined with WebLogo3 (66) sequence logo analysis revealed highly conserved regions (Figs. 3 and 4*C*). These regions were mapped onto structurally aligned proteins and visually inspected to delineate the motif. Independent confirmation was obtained through the MEME suite (67).

### Knotted Solenoid Coordinate Profile.

To assess the β-solenoid architecture, C_*α*_ coordinate profiles were computed as described for RAPHAEL (45), averaging over windows of size 6 and 3 with 200 random translations and rotations. Periodicity quantified from the spacing of local minima (Fig. 4*B*).

### Structural Tree.

To obtain a structure-based tree, we decided to use the FoldTree (68) package. As an input we have prepared a set of experimentally solved structures from the CATH database, supplemented with the AlphaFold predictions of the Knotted Solenoid proteins. Each group was then clustered using PSI-CD-HIT (69, 70) with the threshold of 0.25, 1 representative per cluster with manual verification to ensure the correctness of the input data.

### Expression and Purification of Knotted Solenoid Proteins.

Proteins with UniProtKB IDs: A0A653LYW1 (PDB ID: 9RDS), A0A2W6Z6R8 (PDB ID: 9QIX), A0A1V1V225 (PDB ID: 9SOJ), A0A0Q7SN77 (γ), and A0A4P8QH87 (ϵ) were produced and purified according to the protocol described in *SI Appendix*.

### Crystallization, X-ray Measurements, and Structure Determination.

Diffraction were obtained from crystals grown in the crystallization conditions: 0.1 M sodium acetate pH 4.5, 0.2 M Li_2_SO_4_, 30% w/v PEG 8000 (PDB ID: 9RDS); 0.1 M sodium acetate pH 5.0, 0.05 M LiCl, 15% v/v PEG 400 (PDB ID: 9QIX); and 0.1 M bicine pH 9.0, 25% w/v PEG 3350 (PDB ID: 9SOJ).

Additional information on beamline (78) setup and data processing is available in *SI Appendix*.

### Far-UV Circular Dichroism.

Far-UV circular dichroism (CD) measurements for chemical stability were performed on a BioLogic MOS-450/AF-CD spectrometer with a 0.1 mm pathlength quartz cuvette. Protein samples (50 μM) prepared in SEC buffer (see production and purification section), no glycerol. Measurements at room temperature, covered 210 to 250 nm. Each sample measured twice, 5 s acquisition. Data averaged and smoothed with Savitzky–Golay algorithm. Samples for stability testing with 5 μM protein concentration were prepared in the same conditions with measurements done with a 1 mm cuvette on a Chirascan spectrometer (Applied Photophysics).

Samples for denaturation were equilibrated for 3 h and measured from the lowest chemical denaturant concentration. Samples used for refolding were incubated in 7 M urea for 5 h, then diluted to 50 μM and target denaturant concentrations and equilibrated for 2 h, then measured from the highest denaturant concentration. Optimal concentrations and equilibration times were determined by additional testing (see *SI Appendix* for more details).

Far-UV CD unfolding kinetics were measured with a Chirascan spectrometer and 1 mm pathlength cuvette. Protein samples (5 μM in SEC buffer) mixed with guanidinium chloride (final conc. 4 M) were measured at 25 ^°^C. Kinetics (up to 2,000 s) used 10 s integration per time point.

Thermal stability was measured using a Chirascan spectrometer (Applied Photophysics); 1 mm pathlength cuvette, 10 μM protein, over 20 to 54 ^°^C with five repeats, 0.5 s acquisition. Data were recorded from 200 to 250 nm; lower wavelengths excluded due to high optical density.

### Oligomeric State Analysis.

SEC-MALS and analytical SEC were performed on a Superdex 200 Increase 10/300 GL column (Cytiva). For SEC-MALS, light scattering (LS), ultraviolet absorbance (UV), and differential refractive index (dRI) signals were recorded. Analytical SEC experiments were calibrated with standard protein markers. More details in *SI Appendix*.

### Differential Scanning Fluorimetry.

(DSF) Samples were prepared with increasing protein concentrations from 1 to 11 μM in a SEC buffer, mixed with (10×) SYPRO Orange as the signal dye (Sigma-Aldrich) in 1:1 ratio. Measurements performed on the CFX96 Real-Time PCR machine (Bio-Rad). Two minutes of temperature equilibration at 22 ^°^C, followed by a temperature increase to 95 ^°^C (1 ^°^C/min) with fluorescence intensity (FRET channel) measured. All experiments repeated at least three times for reliability. Data analysis conducted using custom scripts.

### Differential Scanning Calorimetry.

DSC measurements were performed using a MicroCal VP-DSC calorimeter (Malvern, UK). The temperature range between 25 and 90 ^°^C (90 ^°^C/h). Protein samples (50 μM) in SEC buffer. Each protein was measured in at least three replicates.

Analysis of DSC data done with the pyDSC program (71). The program calculates baseline correction following an iterative procedure presented in refs. 72 and 73.

### Molecular Dynamics Simulations and the Generative Deep Learning Model.

The all-atom explicit solvent molecular dynamics simulations were performed in GROMACS 2023.1 software using the CHARMM36 force field. Starting conformations are based on crystal structures (PDB ID: 9RDS and 9QIX). The 200 ns simulations were run independently, 2 times for 9RDS and 4 times for 9QIX structure.

To simulate protein folding and unfolding, we employed a C_*α*_ coarse-grained structure-based model representation (74) based on the SMOG server (75). We use the shadow native contact map (76), Gaussian potential (77). All simulations were conducted in Gromacs v4.5.4 using leap-frog stochastic dynamics with an inverse friction coefficient equal to 1.0 and time step equal to 0.0005 τ.

In the case of dimer simulations, the centers of mass of the subunits were connected by a soft spring to ensure that the proteins did not drift apart. The force constant of this interaction was equal to the force constant of the bonding interaction between beads scaled by 0.00005. First, we performed individual long thermal unfolding trajectories to generate a large number of unfolded conformations for the dimer and monomer. Next, 500 and 300 folding trajectories for a dimer and monomer were executed at $T=1.073\;\epsilon /k_{B}$ (dimer), $T=1.064\;\epsilon /k_{B}$ (monomer), started from randomly chosen topologically trivial and unfolded conformations. In the case of the unknotted solenoid F(Q, RMSD), it was determined at a temperature of $T=1.146\;\epsilon /k_{B}$. The free energy landscape was calculated using the WHAM method.

We also used the generative deep learning model, BioEmu (46). BioEmu was used with standard parameters to generate an ensemble of 10,000 statistically independent structures, which effectively emulated the equilibrium distribution in a fraction of the computational cost (*SI Appendix*, Fig. S19).

### Detection of a Unique Set of Structural Features of Knotted Solenoids.

The complete description with technical details is described in *SI Appendix*. To identify structural features distinguishing Knotted Solenoids from other members of this class, we performed a comprehensive analysis of all biological assemblies deposited in the RCSB PDB and assigned to CATH lineage 2.160. (including all 2.160.X.Y subgroups)

The main β-solenoid channel was defined according to CATH annotations and based on axial rise characteristic of β-solenoids (32) (around 4.5 to 5.1 Å of axial rise). Interchain spatial proximity was assessed by identifying Cα pairs from different chains located within 8 Å with residues then classified into the terminal or central region of the structure.

All dimeric assemblies were subsequently examined manually. During this inspection, we evaluated a number of features, including: the cross-sectional geometry of the solenoidal channel, the relative orientation of chains, the alignment order, overall symmetry, and the hydrophobic character of the interchain contact surface. This combined automated and manual assessment enabled the identification of feature combinations unique to Knotted Solenoids within the analyzed β-solenoid dataset.

## Supplementary Material

Appendix 01 (PDF)

## Data Availability

Data has been provided to the repository under link: https://doi.org/10.58132/3YFCPO (79). All other data are included in the article and/or *SI Appendix*.
