# Supplementary material for: Novel Knotted Solenoid fold with order-shifted coil arrangement leads to nontrivial 31 topology
Source: Proc Natl Acad Sci U S A. 2026 Apr 22;123(17):e2525920123. doi: 10.1073/pnas.2525920123 (PMC13123833; doi:10.1073/pnas.2525920123)
Supplement: Supplementary file 1 — Appendix 01 (PDF) [file pnas.2525920123.sapp.pdf]

Supporting Information for

Novel Knotted Solenoid fold with order-shifted coil arrangement leads to non-trivial 3_1 topology

Maciej Sikora, Mariusz Mozajew, Julia A. Sikorska, Fernando Bruno da Silva, Agata P. Perlinska, Anna Kluza, Szymon Niewieczerzal, Maciej Lukasiewicz, Beata Wielgus-Kutrowska, Karolina Stachurska-Korzeniowska, Sophie E. Jackson and Joanna I. Sulkowska

Corresponding author: Joanna I. Sulkowska
E-mail: j.sulkowska@cent.uw.edu.pl

This PDF file includes:

Supporting text
Figs. S1 to S22
Tables S1 to S3
SI References

Supporting Information Text

Solenoid Table

solenoid_table.xlsx

The table contains the list of all Solenoid Proteins with experimental structure resolved, as well as Knotted Solenoid Proteins with their assignment to the respective CATH ID. The mapping was done via UniProtKB Gene3D annotations. The data were obtained from the 2024_05 version.

Diffraction data statistics

Table S1. Diffraction data collection and refinement statistics of Knotted Solenoid proteins.

Protein PDB ID	9RDS	9QIX	9SOJ
**UniProtKB ID**	**A0A653LYW1**	**A0A2W6Z6R8**	**A0A1V1V225**
**Data collection**			
Beamline	DESY P13	DESY P13	BESSY II 14.1
Space group	$P3_121$	$P6_3$	$P2_12_12$
α, β, γ [°]	90, 90, 120	90, 90, 120	90, 90, 90
a, b, c [Å]	57.7, 57.7, 70.5	133.4, 133.4, 53.4	82.0, 82.2, 88.3
Resolution [Å]	40.76-1.22 (1.29-1.22)	48.46-1.66 (1.76-1.66)	48.51-1.88 (1.99-1.88)
Number of unique reflections	40 919 (6 516)	64 464 (10 291)	49 168 (7 708)
Rmeas [%]	8.2 (158.3)	6.7 (235.4)	23.9 (127.4)
I / δ (I)	13.8 (1.2)	25.4 (1.1)	12.8 (2.3)
CC(1/2)	100.0 (56.7)	100.0 (58.0)	100.0 (75.4)
Completeness [%]	100.0 (100.0)	99.9 (99.4)	99.7 (98.1)
Multiplicity	9.9 (9.5)	20.6 (20.7)	12.8 (12.4)
B(Wilson) [A^2]	19.3	39.8	19.6
Mosaicity [°]	0.11	0.14	0.27
ISa	27.4	25.6	18.5
**Refinement**			
Resolution range [Å]	28.85-1.22	32.04-1.66	38.82-1.90
Completeness for range [%]	100.0	99.8	99.9
Number of reflections	40 915	64 450	47 668
Rwork / Rfree	0.130 / 0.156	0.156 / 0.180	0.163 / 0.211
Number of non-hydrogen atoms	1 229	2 411	4 598
Average B factor [A^2]	20.7	41.7	23.4
**Structure quality**			
**R.m.s deviations**			
Bond lengths [Å]	0.011	0.007	0.007
Bond angles [°]	1.1	0.9	0.9
**Ramachandran statistics**			
Favored [%]	96.1	97.3	97.5
Allowed [%]	3.9	2.7	2.1
Outliers [%]	0.0	0.0	0.4
**Molprobity analysis**			
Clashscore	1.4	2.8	3.4
Poor rotamers [%]	0.0	0.5	0.0
Allowed rotamers [%]	1.8	1.8	3.2
Favored rotamers [%]	98.2	97.7	96.8
Molprobity score	1.13	1.20	1.18

Spectra fitting and parameters

The fitting of the 220 nm ellipticity values for the far-UV CD spectra in varying urea concentrations was performed using a two-state unfolding model equation.

Equation S1:

$$S = \frac{\alpha_N + \alpha_U \cdot e^{m([D]-[D]_{50\%})}}{1 + e^{m([D]-[D]_{50\%})}}$$

Where $[D]$ is the denaturant concentration, $[D]_{50\%}$ is the transition midpoint and m defines slope. α_N and α_U denote the signal from the native and unfolded state. The optimal parameters with the fitting error values in the brackets, as well as overall RMSE, were found to be as follows:

Table S2. Thermodynamic parameters from the best fit of the chemical denaturant-induced unfolding data to a two-state unfolding model. All measurements done in 50 μM with the exception of 9RDS low conc. row conducted in 5 μM (Figure S17).

		α_N (deg · cm^2 / dmol)	α_U (deg · cm^2 / dmol)	m (kcal / mol · M)	$[D]_{50\%}$ (M)	RMSE
9RDS	unfolding	-9.44×10^5 (4.14×10^3)	-4.97×10^5 (4.42×10^3)	3.31 (0.18)	4.62 (0.02)	1.1×10^4
	refolding	-9.30×10^5 (5.63×10^3)	-4.99×10^5 (4.66×10^3)	3.89 (0.26)	4.66 (0.02)	1.2×10^4
	low conc.	-9.7×10^5 (1.33×10^3)	-3.93×10^5 (2.04×10^3)	1.78 (0.2)	4.28 (0.07)	2.9×10^3
9QIX	unfolding	-9.18×10^5 (5.83×10^3)	-4.86×10^5 (7.19×10^3)	3.32 (0.28)	4.89 (0.03)	1.6×10^4
	refolding	-9.17×10^5 (8.47×10^3)	-4.86×10^5 (8.09×10^3)	3.84 (0.42)	4.88 (0.03)	1.8×10^4
ϵ	unfolding	-9.39×10^5 (5.16×10^3)	-5.00×10^5 (6.09×10^3)	4.12 (0.35)	4.91 (0.02)	1.4×10^4
	refolding	-9.43×10^5 (6.34×10^3)	-4.87×10^5 (6.31×10^3)	3.51 (0.26)	4.92 (0.03)	1.4×10^4

Values collected at 220 nm from far-UV CD kinetics for protein 9RDS were fitted to a single-exponential function:

$$S(t) = c + a \cdot e^{-kt}$$

where t (sec.) is the independent variable signifying time, a (a.u.) is the amplitude of the exponential decay, c (a.u.) is the baseline offset representing residual signal at long times, and k (s^{-1}) is the rate constant of the unfolding process.

Fitting parameters with errors and the half-life $t_{1/2} = \ln 2/k$ for each replicate and the averages are shown in the table below.

Table S3. Far-UV CD kinetic parameters and half-lives obtained from single-exponential fits for protein 9RDS.

Replicate	a (a.u.)	k (s^{-1})	$t_{\frac{1}{2}}$ (s)
1	2.42 ± 0.06	0.02 ± 0.001	31.4 ± 1.0
2	2.33 ± 0.16	0.04 ± 0.003	19.3 ± 1.6
3	2.28 ± 0.08	0.03 ± 0.001	26.1 ± 1.2
Averaged	2.34 ± 0.07	0.03 ± 0.007	25.6 ± 6.1

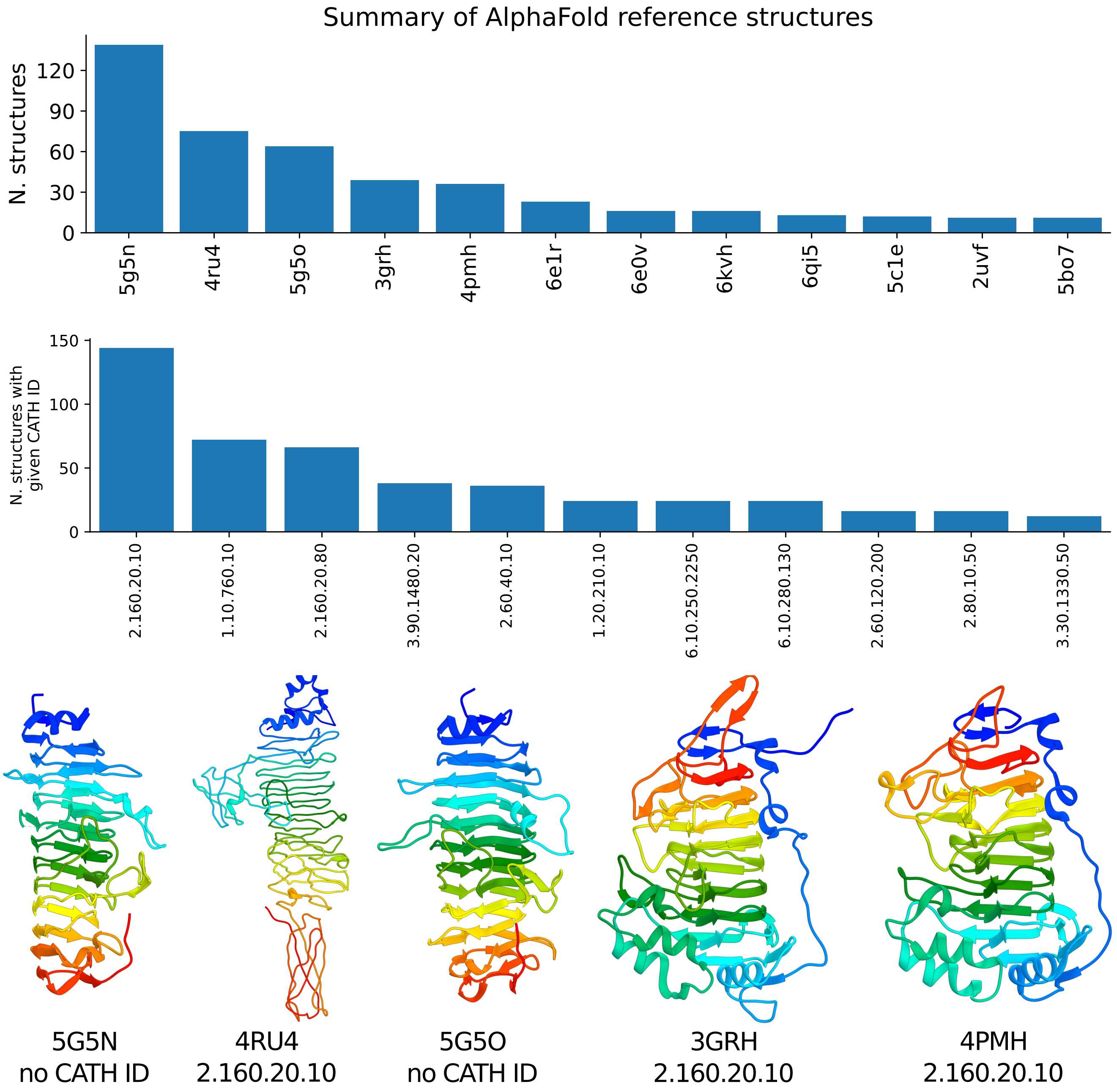

Fig. S1. Overview of the template structures used by the AlphaFold model to predict all of the structures of the knotted proteins with the Solenoid fold. Statistics include both PDB structures as well as their assigned CATH IDs, with the most frequent representatives visualized.

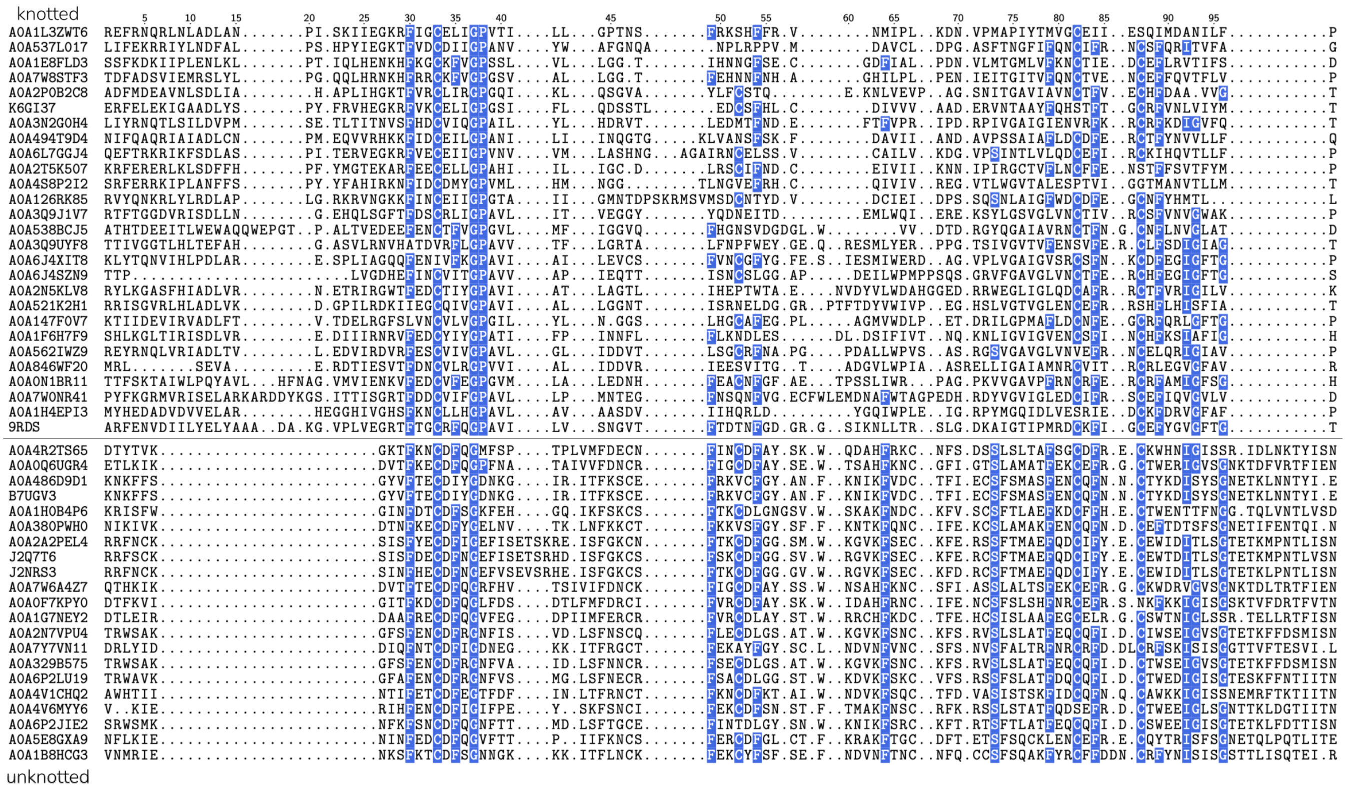

Fig. S2. Alignment of knotted and unknotted solenoids. 26 representatives of Knotted Solenoids and protein 9RDS, and 21 unknotted solenoids that are sequentially closest to the knotted ones. Highlighted positions represent especially conserved positions.

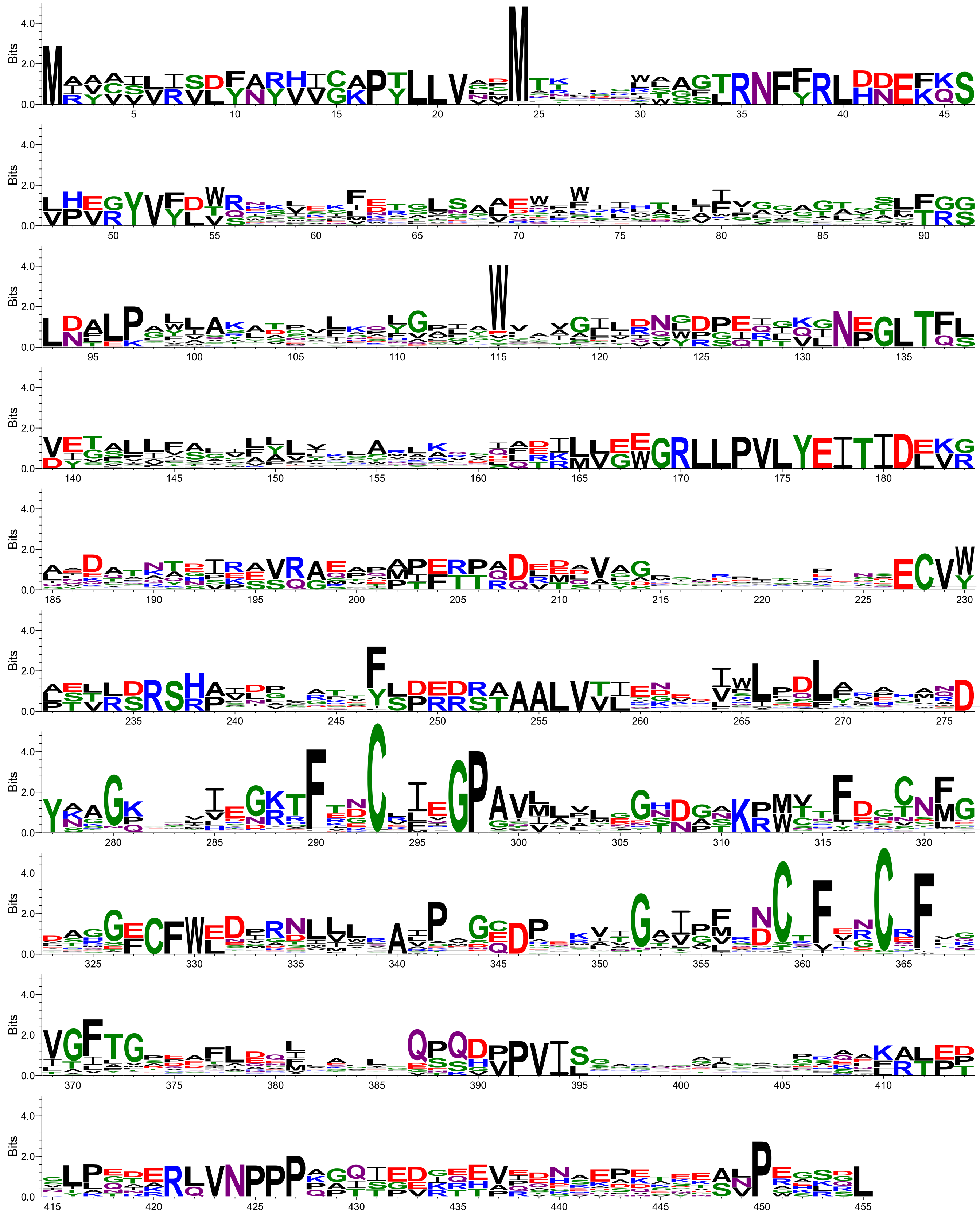

Fig. S3. Sequence logo of the Knotted Solenoid proteins with the 3_1 knot. The sequence alignment was made using the MAFFT service, and the sequence logo was created using the WebLogo 3 website.

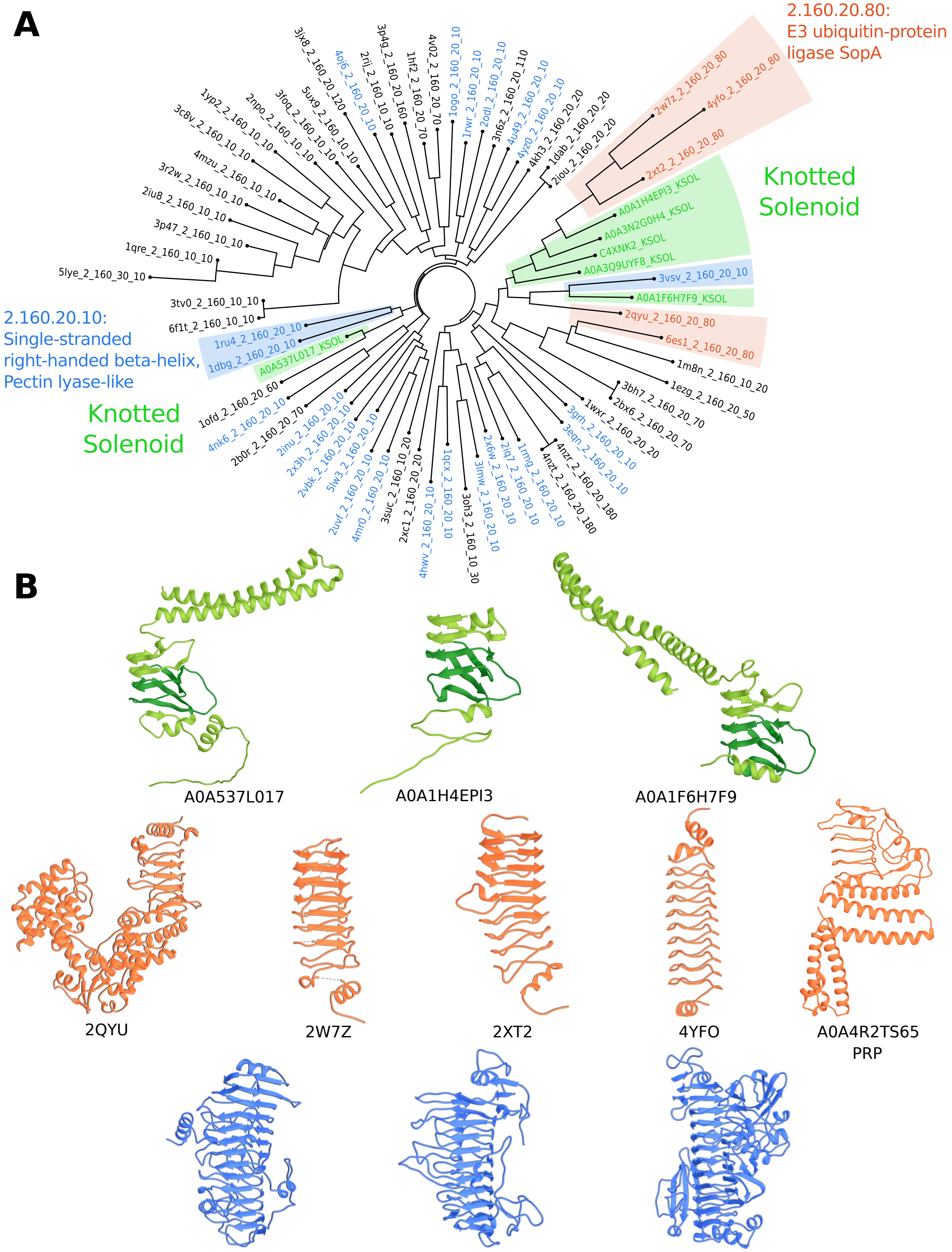

Fig. S4. A: Structural-based tree created with the FoldTree package. B: Example structures from the tree close to Knotted Solenoids. Additionally added the PRP structure for comparison. As input, we've prepared a set of experimentally resolved structures from the CATH Database and supplemented them with the AlphaFold predictions of the Knotted Solenoid proteins. Each group was then clustered using PSI-CD-HIT with a threshold of 0.25. We picked only the first chain with the Solenoid fold from each structure. The tree presents a different perspective, showing structural similarity relations between solenoids and confirming that groups with CATH ID: 2.160.20.10 and 2.160.20.80 are consistently structurally the closest groups to the novel Knotted Solenoid fold.

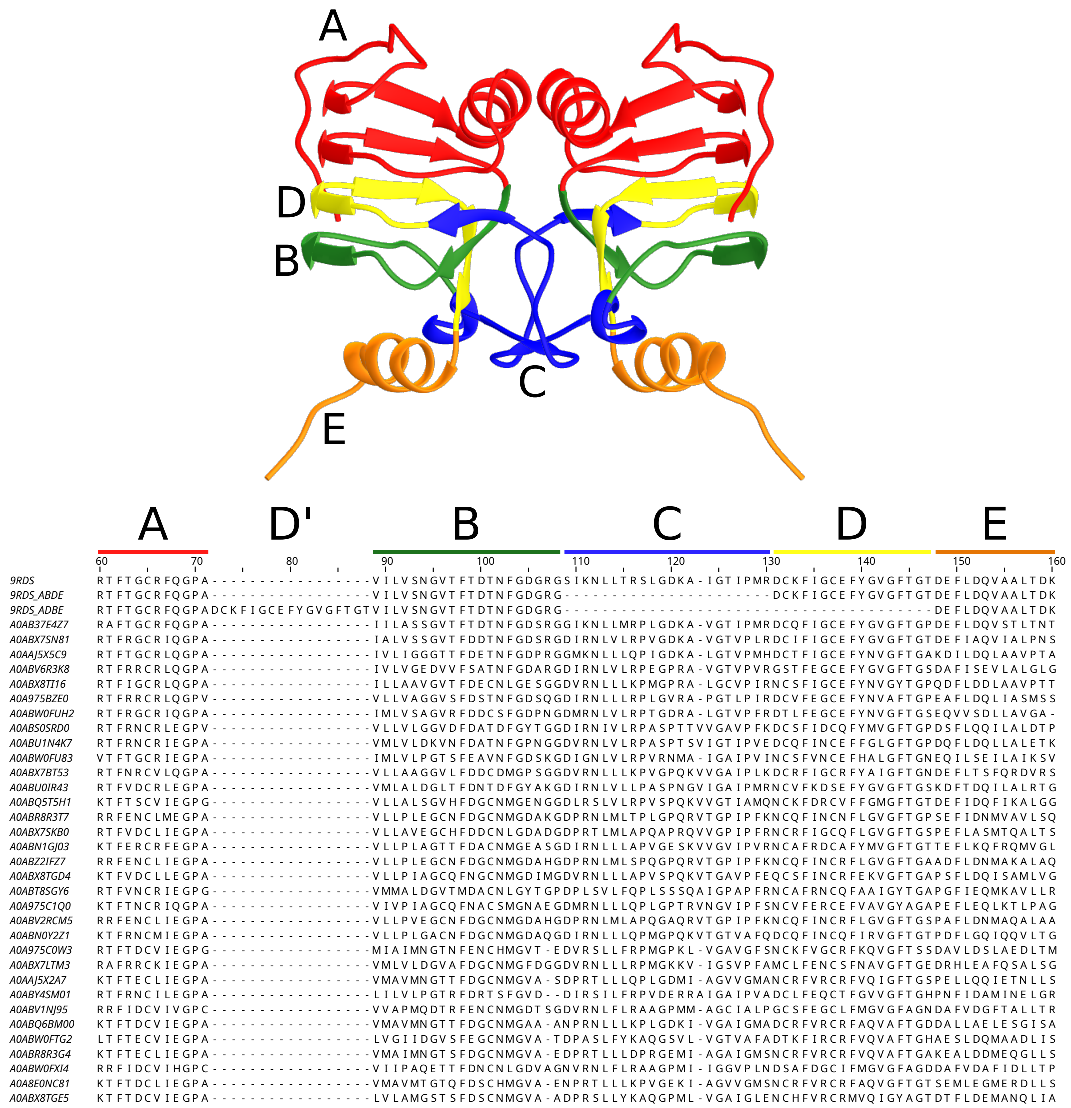

Fig. S5. Example of splitting Knotted Solenoid into sections for evolutionary analysis. Sections include, in order: N-terminal section and first two loops (red), skipping loop (green), backtracking loop (blue), threading loop (yellow), C-terminal section (orange). Below the structure, an alignment is shown for structures with no AlphaFold prediction against native Knotted Solenoid 9RDS, as well as constructs: 9RDS_ABDE (only removed backtracking loop) and 9RDS_ADBE (removed backtracking loop and reversed order of loops). All proteins align closely with native 9RDS.

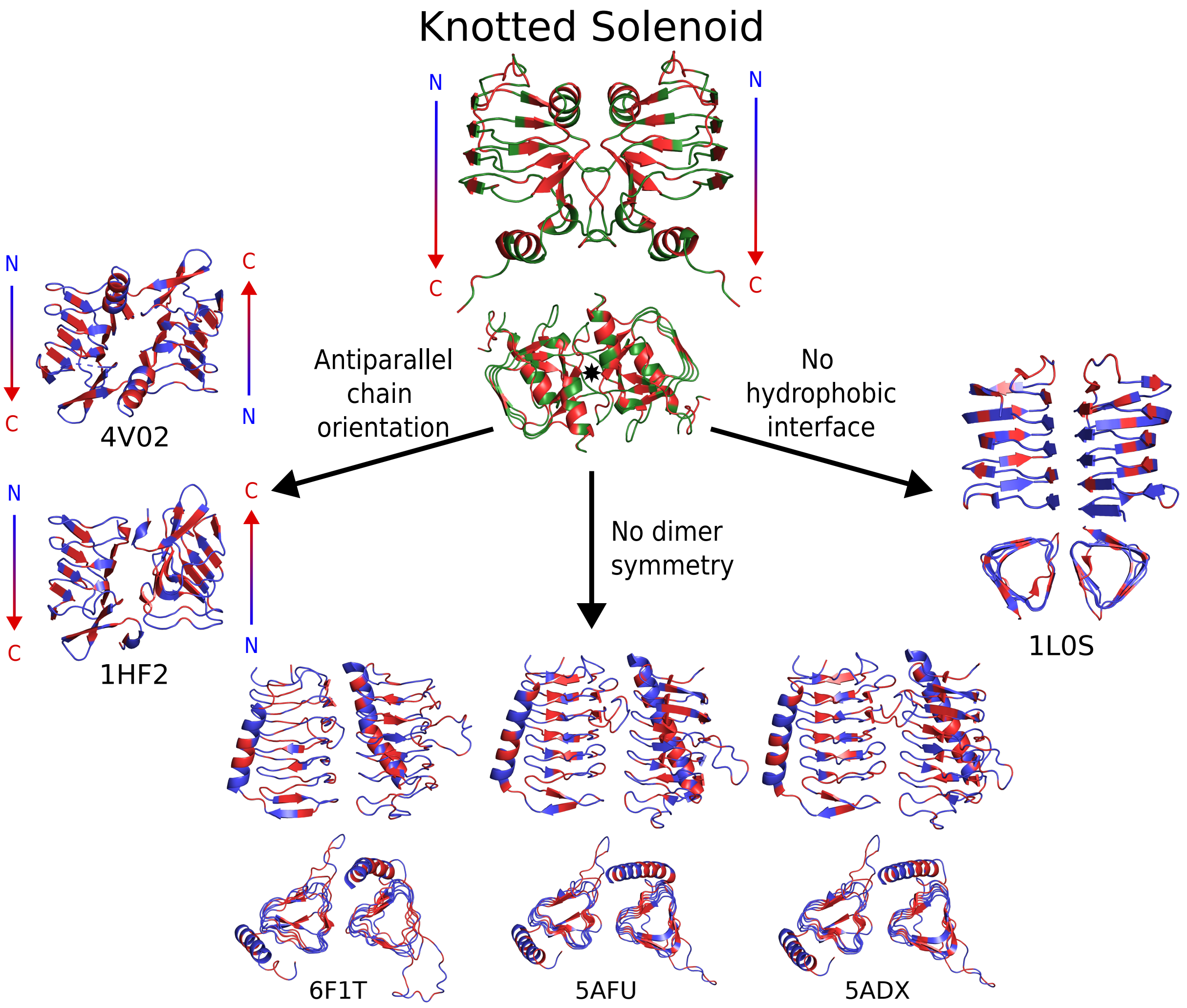

Fig. S6. Comparison between Knotted Solenoid fold and selected other members of β-solenoids. While Knotted Solenoids belong to the broader group of β-solenoids, they can be distinguished by a set of features: It forms a dimer with triangle-shaped channels with parallel chain (N-C) orientation, and a hydrophobic interface between chains. The figure shows examples of other β-solenoids that are lacking at least one of the aforementioned characteristics while still forming dimeric assemblies with a triangular channel shape. In red, marked hydrophobic residues.

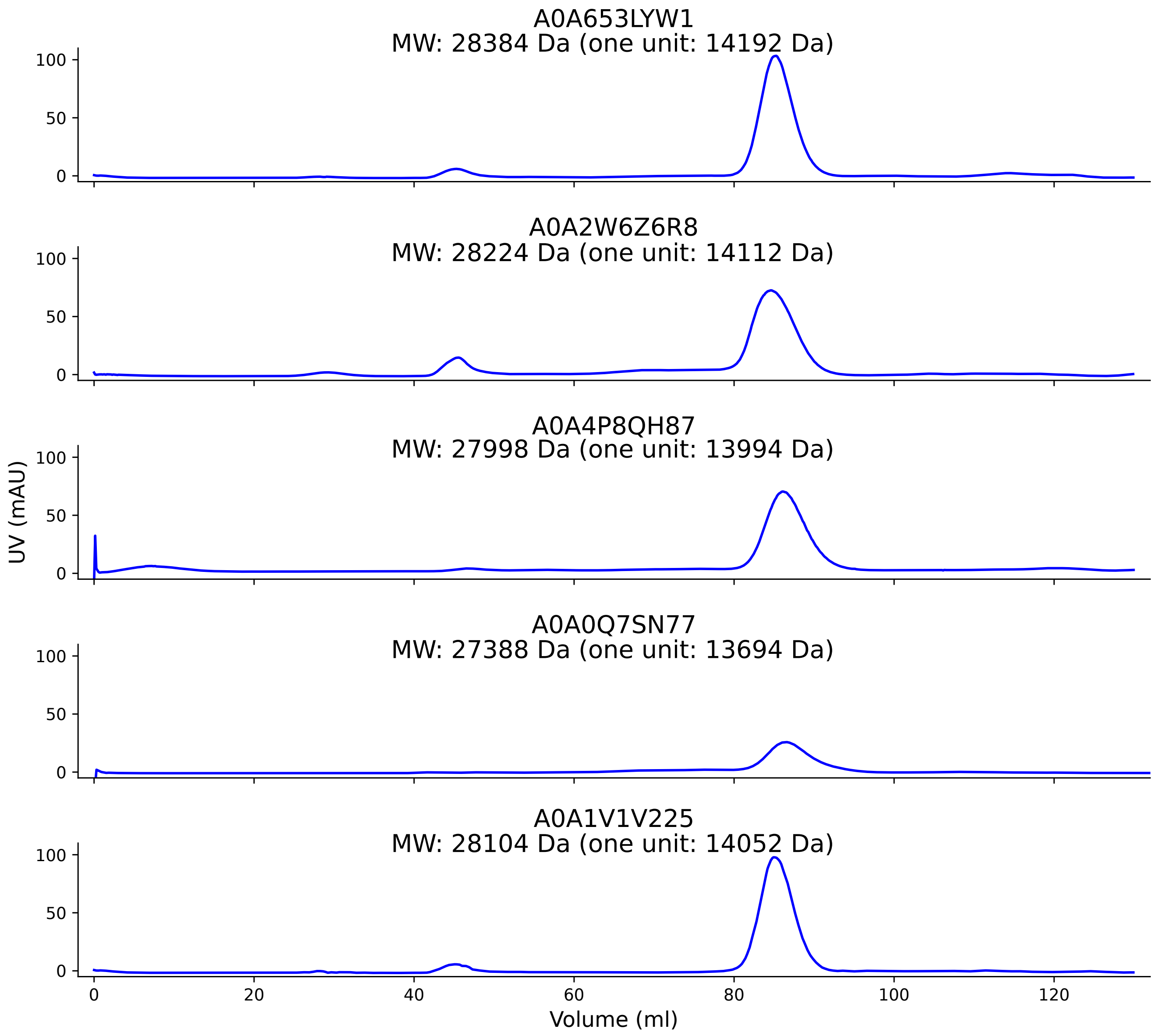

Fig. S7. FPLC UV chromatograms obtained during the final purification process of the Knotted Solenoid proteins. Fractionation was performed on the ÄKTAxpress chromatography System with a HiLoad Superdex 16/600, 200 pg Column by Cytiva. The protein peaks appeared between 80 and 90 ml with the approximate molecular weight confirmed by SDS-PAGE.

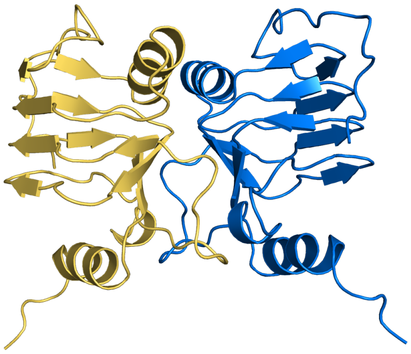

Fig. S8. Knotted Solenoid 9RDS (yellow) shown together with a crystallographically related mate (blue) generated from the space-group symmetry. The two copies form a dimeric structure similar to those reported for Knotted Solenoid 9QIX and 9SOJ.

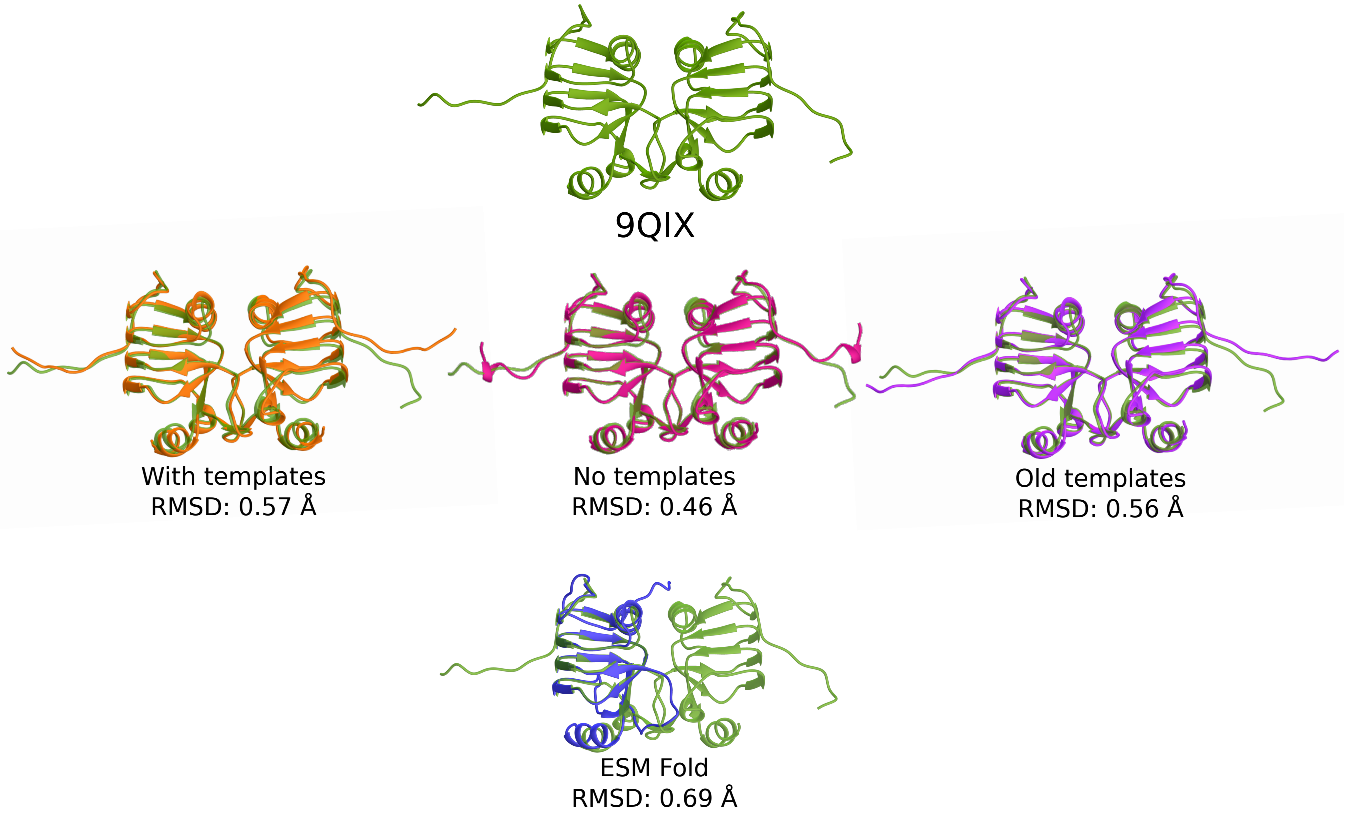

Fig. S9. Comparison of crystal structure 9QIX with AlphaFold 3 and ESM Fold predictions with RMSD values (pruned atom pairs). In order from left: prediction conducted with default template settings, prediction with templates turned off, prediction with templates dated up to 01.01.1995 with a limited pool of suitable templates. Below additional prediction with ESM Fold model. All predictions made on a single sequence of 9QIX, not multiple sequence alignments.

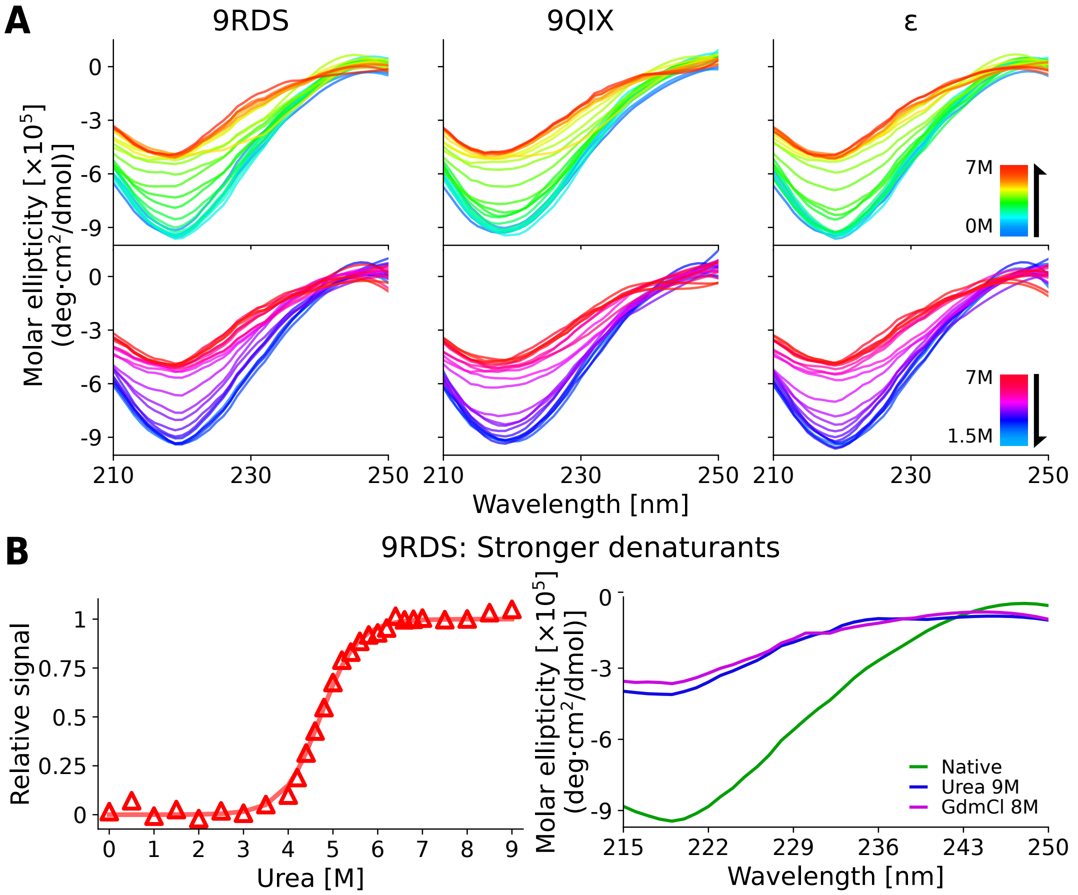

Fig. S10. A: Far-UV circular dichroism spectra for chemical denaturation (first row) and renaturation (second row) of proteins 9RDS, 9QIX and ϵ. B: Far-UV CD measurements results obtained for protein 9RDS using stronger denaturants. On the left side, the fitting of the samples for urea up to 9 M. On the right side, comparison of spectra between the native state and samples in 9 M of urea, and 8 M of guanidinium chloride. All the measurements were performed on the Bio-Logic MOS-450 CD Spectrometer using a 0.1 mm quartz glass cuvette (106-QS, HP Quartz Glass by Hellma Analytics) at room temperature (samples were kept on ice prior to measurement with the exception of urea above 7 M and guanidinium chloride to avoid crystallization). Samples were prepared in 50 μM concentration in buffer containing 50 mM Tris pH 7.4 at 25 $^{\circ}$C and 200 mM NaCl, no glycerol.

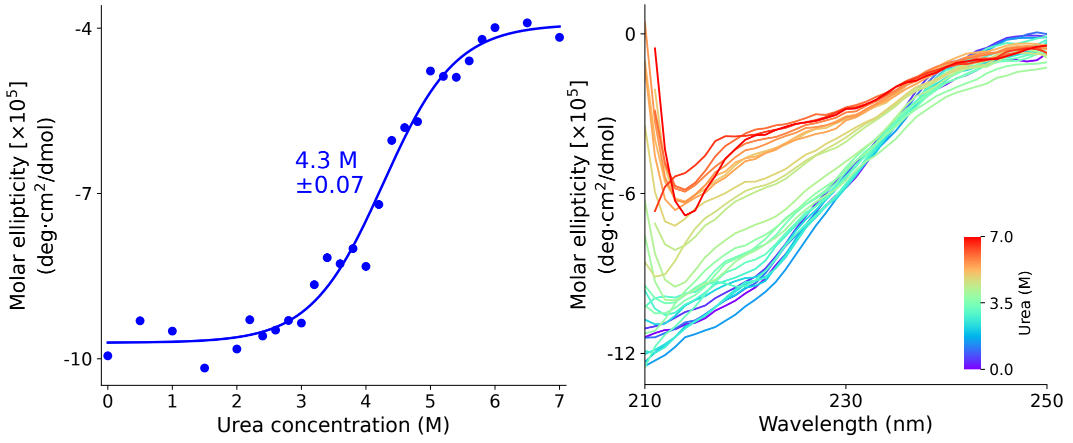

Fig. S11. Far-UV CD measurements in low protein concentration. Unfolding curve showing the molar ellipticity at 220 nm as a function of urea concentration. The solid line represents the fit to a two-state unfolding model, with the midpoint of denaturation indicated together with the associated fitting error. On the right, smoothed far-UV CD spectra recorded at selected urea concentrations during the unfolding transition. All measurements were carried out on a Chirascan (Applied Photophysics) spectrometer with a 1 mm quartz glass cuvette at room temperature. Samples were kept on ice prior to acquisition. Protein concentration was 5 μM in buffer containing 50 mM Tris (pH 7.4 at 25 ^{o}C) and 200 mM NaCl, without glycerol.

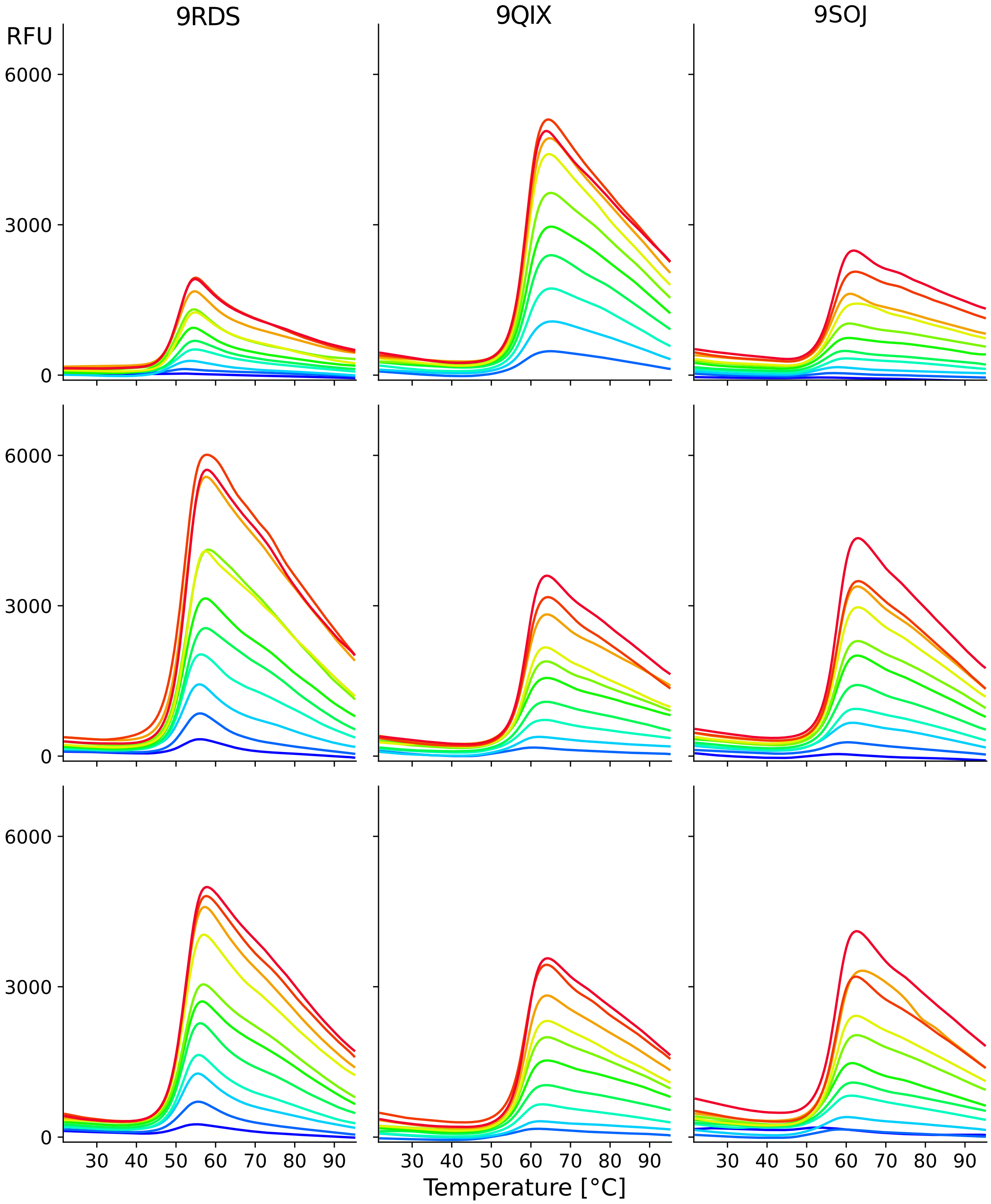

Fig. S12. Repeats of the DSF measurements for proteins 9RDS, 9QIX and 9SOJ. Color gradient represents the concentration of the sample, with blue being the lowest (1 μM) and red being the highest (11 μM), every 1 μM. Protein samples were prepared in SEC buffer and mixed with (10x) SYPRO Orange as the signal dye ina 1:1 ratio and measured on a CFX96 Real-Time PCR machine (Bio-Rad).

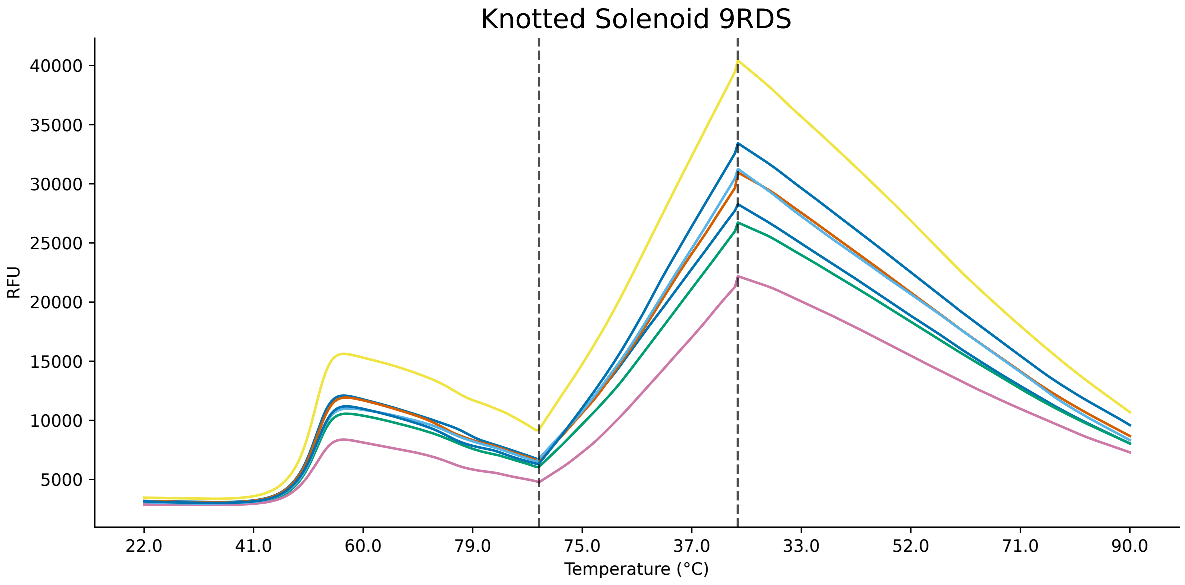

Fig. S13. Repeats of the DSF measurements for protein 9RDS. Each curve represents an independent sample at the same concentration of 11 μM. The full procedure consists of the following phases, marked on the plot: initial heating from 22 to 90 $^\circ$C, cooling from 90 to 22 $^\circ$C, and a second heating from 22 to 90 $^\circ$C. The analysis indicates that the thermal unfolding is irreversible. Protein samples in SEC buffer were mixed with (10x) SYPRO Orange as the signal dye in a 1:1 ratio and measured on a CFX96 Real-Time PCR machine (Bio-Rad).

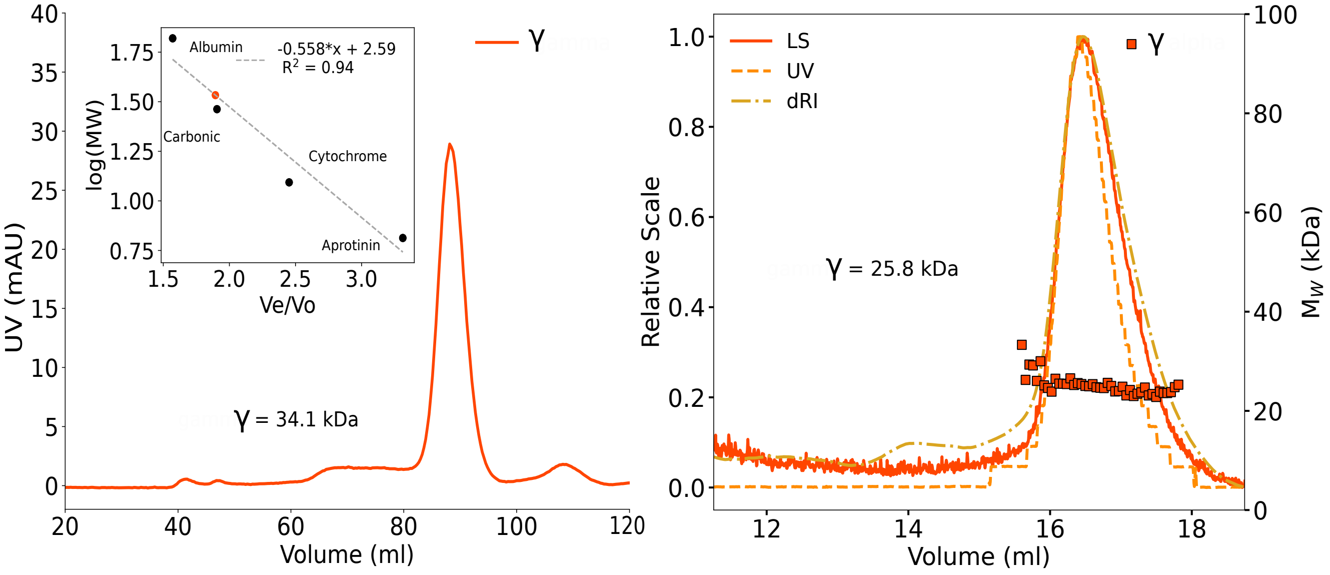

Fig. S14. Characterization of the size and compactness of Knotted Solenoid γ under native conditions. Left panel: The chromatogram displays the elution profile obtained during FPLC purification for Knotted Solenoid γ. The inset shows the calibration curve (in the Methods section) used to estimate the molecular weight (MW) of the proteins. Right panel: SEC-MALS analysis of native protein Knotted Solenoid γ. The graph compares the light scattering (LS), ultraviolet absorbance (UV), and differential refractive index (dRI) signals. Data were normalized to a 0-1 range by dividing each chromatogram by its maximum value across the elution volume. Samples of native and refolded proteins at a concentration of 2 mg/ml were loaded onto a Superdex 200 Increase 10/300 GL column (Cytiva) pre-equilibrated with SEC buffer at a flow rate of 0.75 ml/min using an ÄKTA Pure chromatography system (Cytiva).

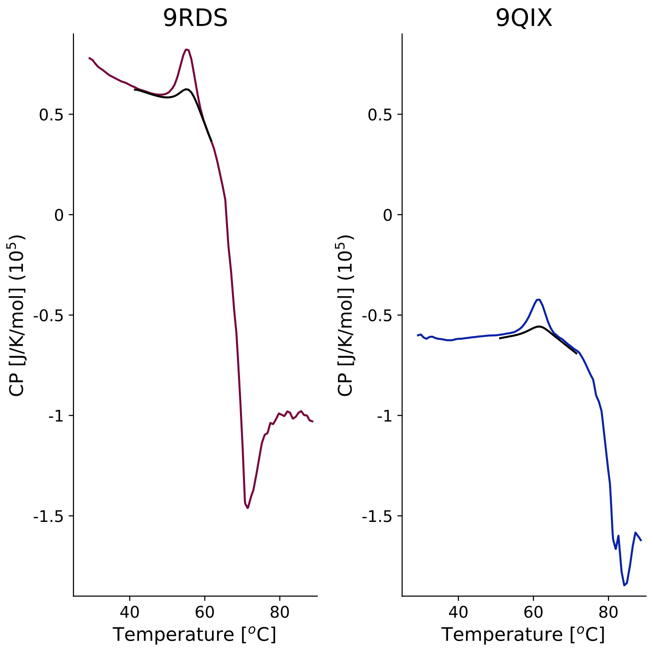

Fig. S15. Differential scanning calorimetry (DSC) was performed to characterize the thermal unfolding profiles of the proteins with Knotted Solenoid fold. The heat capacity was recorded as a function of temperature (^{o}C) for each protein, with two independent replicates per sample. With the black line, a baseline is shown fitted in the peak region to allow for proper thermodynamic analysis. The thermal unfolding was shown to be irreversible, such that only estimates of thermodynamic parameters can be made. Protein samples were prepared in 50 μM concentration in SEC buffer.

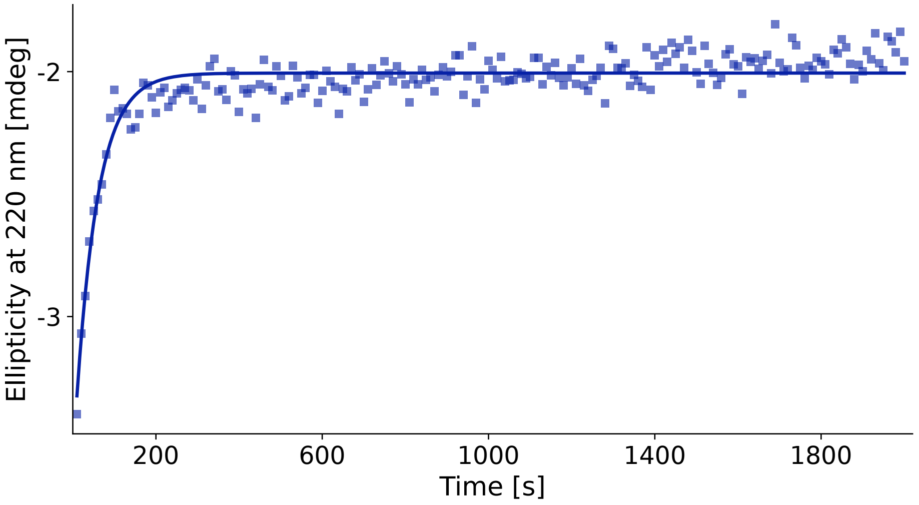

Fig. S16. 2 000 seconds run of far-UV CD unfolding kinetics (220 nm) of protein 9RDS mixed with guanidinium chloride (4 M final concentration) with corresponding single-exponential fit. Results show no additional phases after initial unfolding.

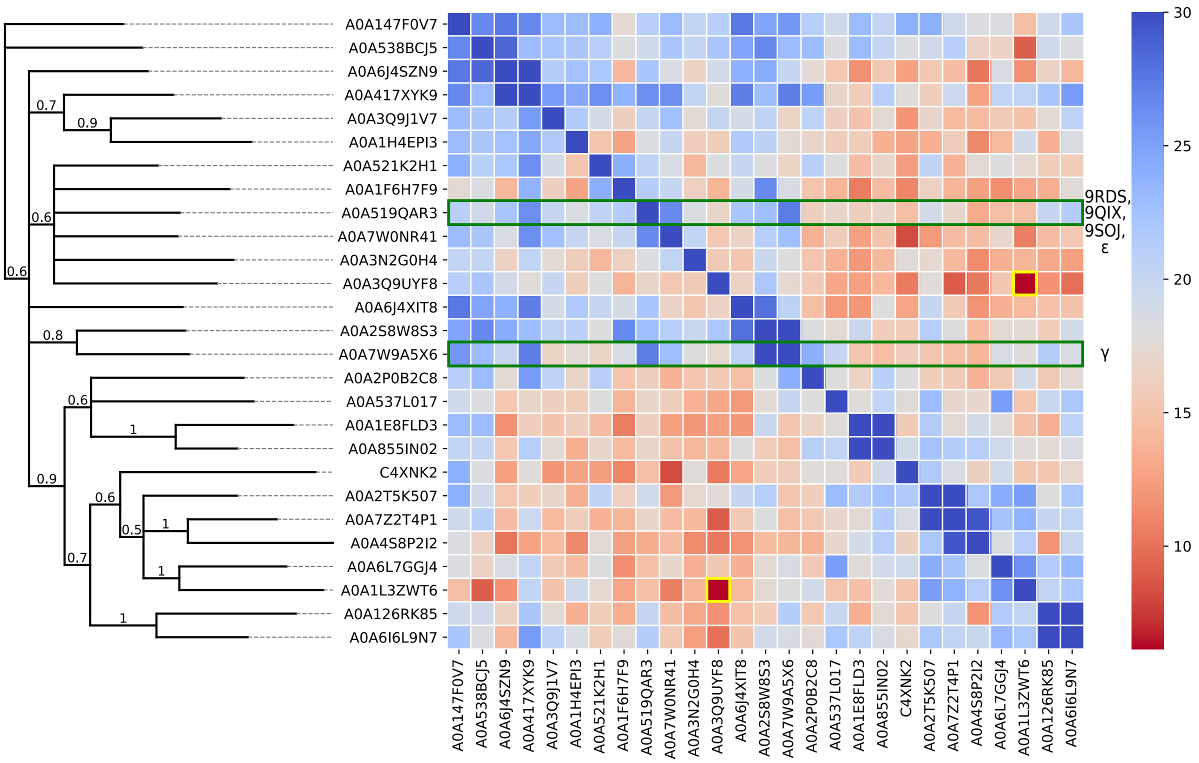

Fig. S17. The comparison of representative proteins with Knotted Solenoid fold (at most 30% sequence identity). The color gradient represents the identity level, with blue being the highest and red the lowest. The least similar protein pair is A0A3Q9UYF8 and A0A1L3ZWT6 UniProtKB ID (6.1% sequence identity). Green rectangles highlight representatives with the highest similarity to the purified proteins. Additionally, on the left of the matrix, a bayesian tree is presented with the probability values on the internal branches.

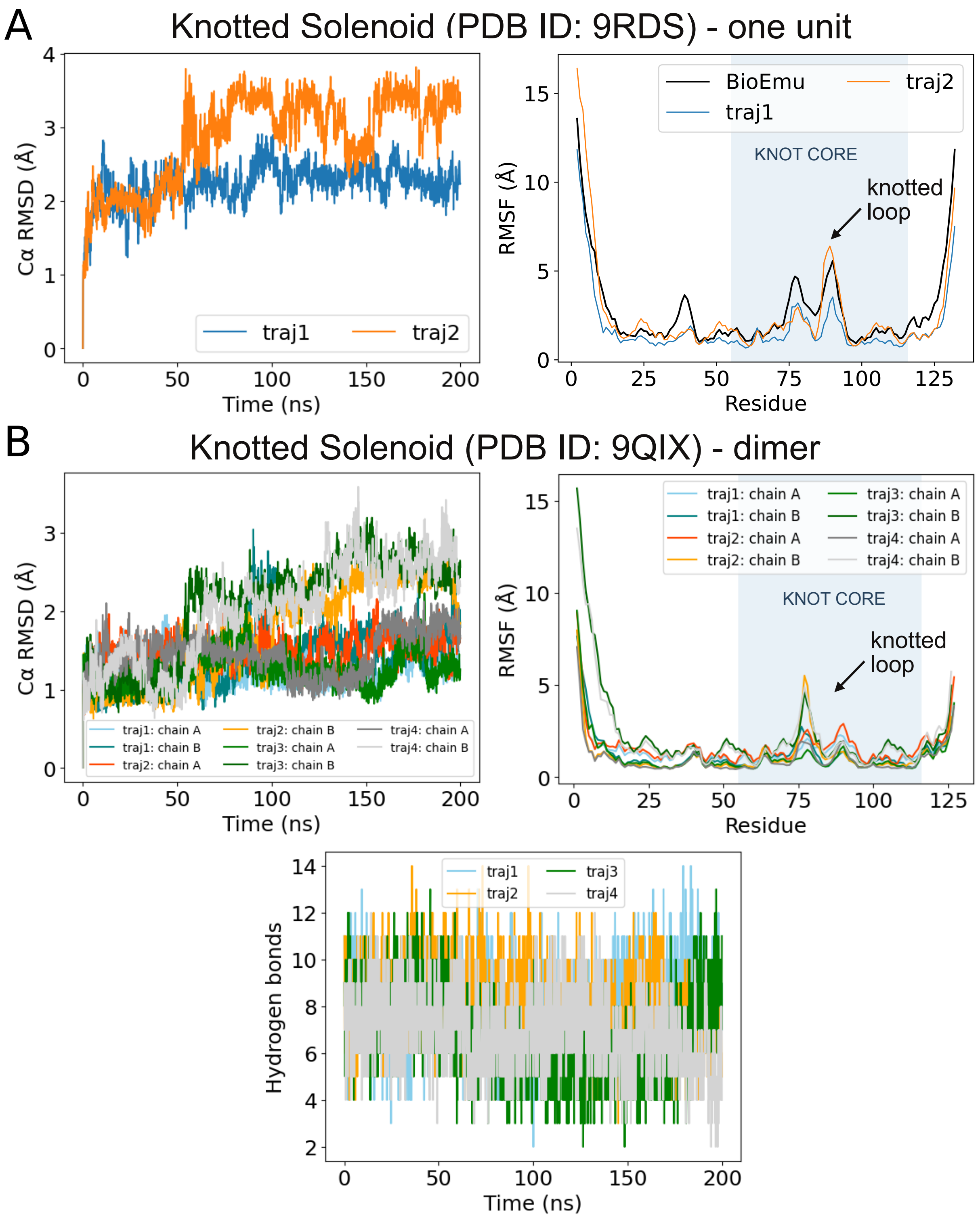

Fig. S18. Characteristics of local stability based on all-atom simulations in explicit solvent and the BioEmu approach, using Knotted Solenoids crystal structures. A: RMSD as a function of time (left panel) and RMSF for each residue (right panel) for one chain of protein 9RDS based on all-atom simulations. B: Simulations for the dimer, protein 9QIX. The RMSF plot shows high flexibility of the N- and C-terminal ends for both the simulated monomer and dimer, in both the all-atom and BioEmu model (black solid line), as expected. Therefore, in the RMSD plot the values are shown for truncated proteins: residues 12–128 for 9RDS and residues 12–127 (chain A) and 12–126 (chain B) for 9QIX. The bottom plot shows the number of hydrogen bonds over time in the protein's dimeric interface based on all-atom model data from panel B.

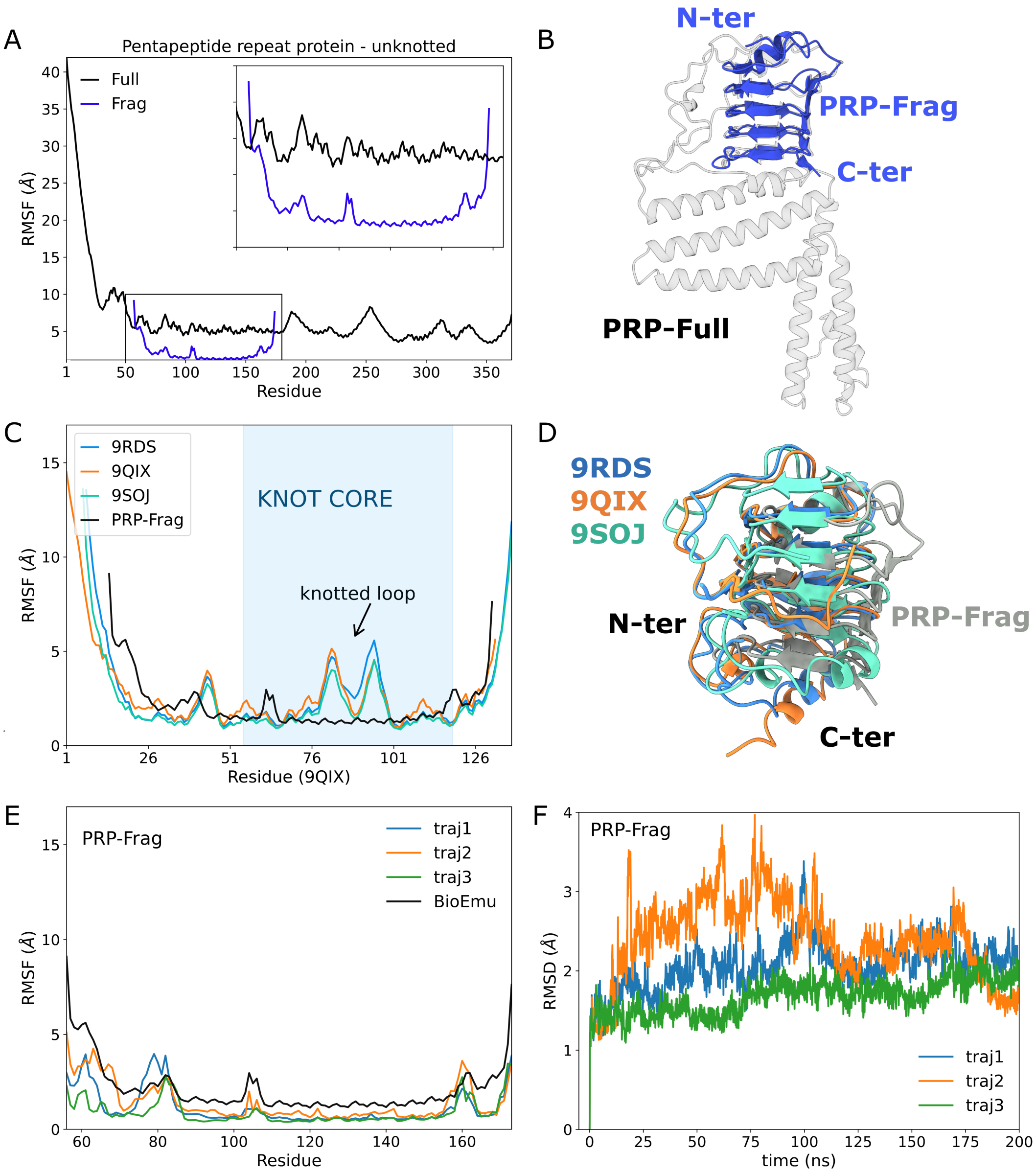

Fig. S19. A: Root-mean-square fluctuation (RMSF) profiles from BioEmu simulations of the unknotted pentapeptide-repeat protein (PRP), comparing the full-length sequence (black solid line) with the fragment (PRP-Frag, blue solid line). The zoomed region highlights how the flexibility of PRP-Frag differs from that of the full-length protein. B: Superposition of the representative structures obtained by BioEmu, comparing PRP-Full (grey) and PRP-Frag (blue). C: RMSF profiles from BioEmu simulations for the Knotted Solenoids 9RDS, 9QIX, and 9SOJ, together with PRP-Frag (Residues numbers are aligned to 9QIX). In proteins with a solenoid motif, fluctuations in the knot are observed, associated with loop fluctuations; these fluctuations do not occur in unknotted solenoids, PRP protein. D: Superposition of the representative structures obtained by BioEmu, comparing the Knotted Solenoids 9RDS, 9QIX, and 9SOJ with PRP-Frag. E: RMSF plot for individual residues in the PRP-Frag protein obtained based on the BioEmu approach and full-atom simulations in an explicit solvent. F: RMSD plot as a function of time (3 trajectories) for the PRP-frag protein based on full-atom simulations in an explicit solvent.

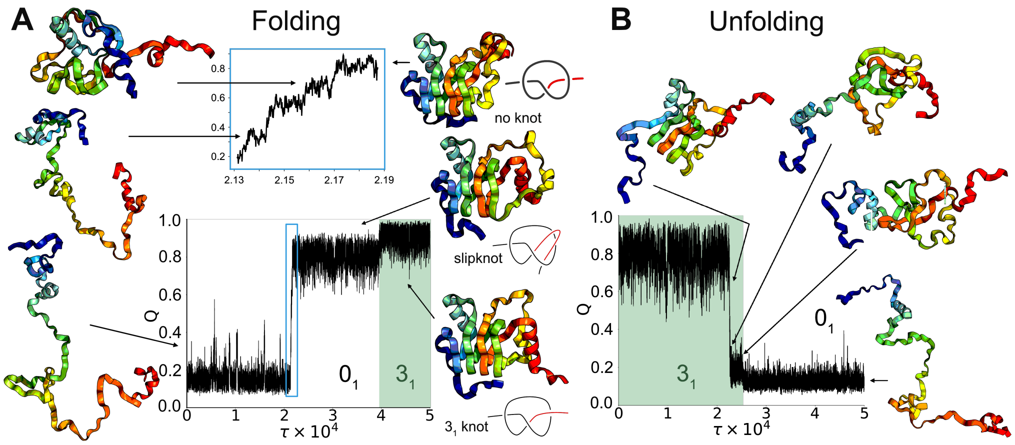

Fig. S20. Folding and unfolding process of the Knotted Solenoid protein. A: Example of a representative folding trajectory. The graph presents the fraction of formed native contacts as a function of time. The inset graph shows the progress of folding in the region marked by a blue rectangle in order to present the progress of the process in more detail, since in this stage, the protein quickly adopts most of its native arrangement. Conformations surrounding the chart are the snapshots from the trajectory. Additional schemes at the conformations on the right side of the graph indicate an embedded entanglement. B: Example of a representative unfolding trajectory. A graph of formed native contacts as a function of time, together with snapshots, shows the progress of the unfolding mechanism.

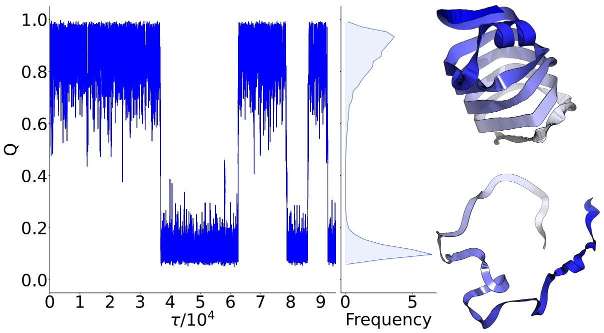

Fig. S21. Folding and unfolding process of the unknotted β-solenoid protein PRP, the closest homolog of the Knotted Solenoid. The figure shows a representative trajectory, including the frequency histogram of the Q values and selected conformations of the folded and unfolded states. The plot presents the fraction of native contacts (Q) as a function of time. In contrast to the Knotted Solenoid, PRP exhibits a simple two-state folding mechanism without detectable intermediates.

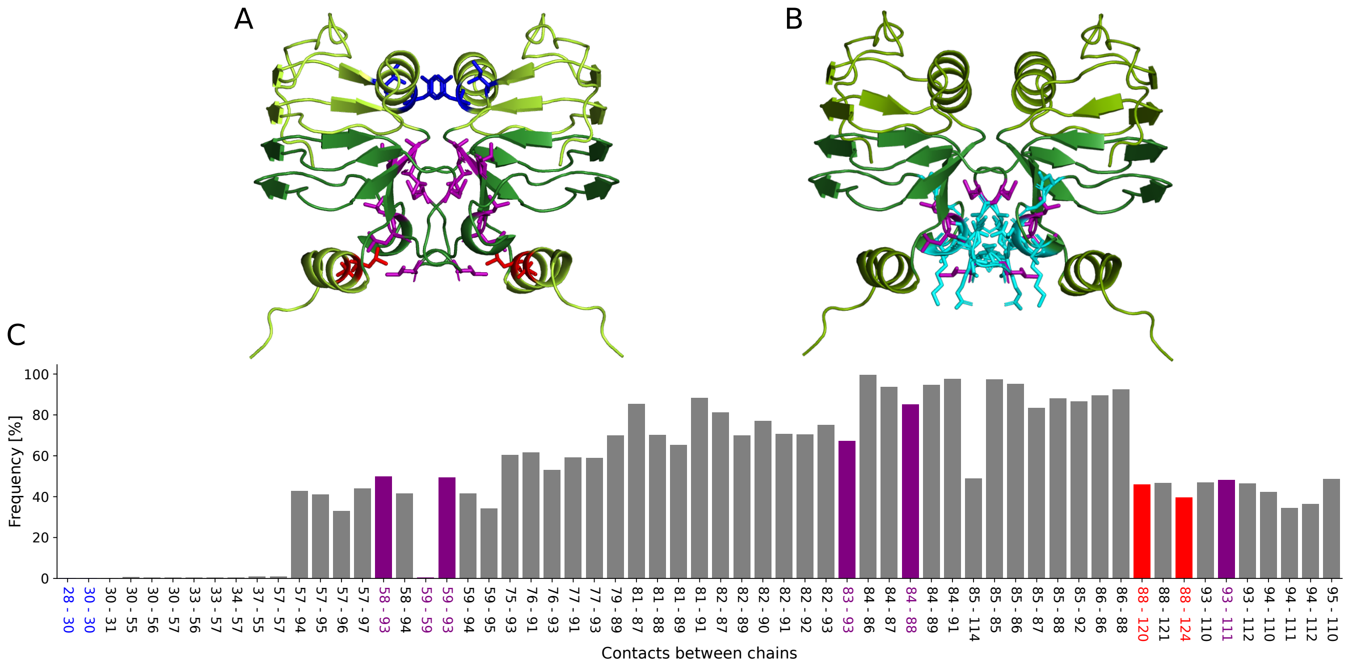

Fig. S22. Analysis of contacts between subunits in their native state and after folding of one of the proteins based on the Knotted Solenoid 9RDS. In total, we identified 61 contacts (based on all-atom shadow map) between chains with 10 forming hydrophobic contacts. Contacts formed with both residues before the knot core include 28–30 and 30–30. Contacts with both residues located within the knot core include 58–93, 59–59, 59–93, 83–93, 84–88, and 93–111. Contacts involving one residue within the knot core and one after the knot core include 88–120 and 88–124. A: The structure of the dimer, with hydrophobic contacts marked in different colors and the dark green area indicating the knot core. Hydrophobic residues forming interchain contacts before the knot core are shown in blue, within the knot core in purple, and after the knot core in red. B: Results of contact frequency in molecular dynamics. The most frequent contacts (over 60 %) between subunits, when 50% of contacts have already been established, after the second chain has been rolled up to its native state. Residues in purple are forming hydrophobic contacts between chains while cyan color highlights the remaining side chains forming contacts. C: Histogram of the frequency of interchain interactions in snapshots where 50% of contacts are formed (after folding other chain) based on molecular dynamics simulations.

Topology determination All structures with the new knotted fold were collected from the AlphaKnot 2.0 database (1, 2) and evaluated. To confirm a knot formed by a protein chain, the projection on a plane is created, and a polynomial knot invariant, such as the Alexander polynomials (3), is computed. This is done in a probabilistic fashion, as proteins are defined as open chains. To close the chain, the endpoints are connected via points on a sphere enclosing the structure. The position of said points will determine the resulting topology; thus, we repeat this evaluation for a large number of equally distributed points on the sphere. Finally, the fraction of connections is assigned as a probability of each resulting knot type. This procedure was followed by applying the Topoly package (4).

Homolog search and motif determination After the identification of the first two proteins (UniProtKB ID: A0A7W8HX03 and A0A2X1AF08), FoldSeek (5) was employed on the AlphaFold representative database (afdb50, afdb-proteome, and afdb-swissprot) and PDB to detect structural homologs. Subsequently, a multiple sequence alignment (MSA) was generated using MAFFT (6), which was then used with HMMER (7) to perform sequential searches across the entire AlphaFold database, applying an e-value cutoff of 10^{-3}.

To identify the common motif in the Knotted Solenoid protein group, both sequential and structural alignments were performed. Sequential alignment using the MAFFT package (8) combined with WebLogo3 (9) sequence logo analysis revealed highly conserved regions. These regions were mapped onto structurally aligned proteins and visually inspected to delineate the motif. Independent confirmation was obtained through analysis with the MEME suite (10).

Expression and purification of Knotted Solenoid proteins Five genes of Knotted Solenoids (UniProtKB IDs: A0A0Q7SN77, A0A653LYW1, A0A2W6Z6R8, A0A1V1V225, A0A4P8QH87) were optimized for expression in *Escherichia coli* by GeneArt (Thermo Fisher Scientific) and cloned into pET51/D-TOPO. The constructs included N-terminal 6xHis-tag cleavable by TEV protease. Vectors were transformed into *E. coli* BL21(DE3) strain for expression.

The LB medium with 100 μg/ml ampicillin was inoculated with single colonies of bacterial cells containing plasmids and incubated overnight at 37 °C, 180 rpm. The overnight cultures were transferred into 2 l of LB medium supplemented with 0.4% glycerol and 100 μg/ml ampicillin, and incubated at 37 °C with shaking until the optical density at 600 nm reached the values of 0.7 - 0.9. The production of target proteins was induced with isopropyl β-D-1-thiogalactopyranoside (IPTG) at a final concentration of 250 μM, then the bacterial cultures were incubated overnight at 16 °C with shaking.

Cells were harvested by centrifugation at 4 200 rpm for 40 minutes at 4 °C using a Multifuge X Pro Series centrifuge by Thermo Fisher Scientific and resuspended in lysis buffer (50 mM Tris pH 8.0 at 4 °C, 500 mM NaCl, 10% glycerol, 10 mM imidazole). Cell lysis was performed by sonication (10 minutes, pulsation ON for 30% of 1 s, 70% amplitude power). Cell

debris was removed by centrifugation at 40 000 rcf for 30 minutes at 4 °C, and the supernatants were collected.

The first step of purification included Ni-affinity chromatography using gravity-flow columns packed with 3 ml of Ni-NTA Superflow resin each (Qiagen) equilibrated with lysis buffer. After loading the columns with the supernatants, the resins were washed with 10 CV of wash buffer (lysis buffer with imidazole concentration increased to 30 mM). Proteins were eluted with the elution buffer (increased imidazole to 300 mM) and fractions pooled based on the absorbance at 280 nm. To remove the 6xHis-Tags, the 6xHis-TEV protease was added to the elution fractions containing proteins and dialyzed overnight (dialysis buffer: 50 mM Tris, 250 mM NaCl, and 5% glycerol, pH 8.0 at 4 °C). The protein samples were once again transferred into Ni-NTA columns to separate the proteins of interest from 6xHis-Tags and 6xHis-TEV protease.

The second purification step involved a size-exclusion chromatography performed with the use of a Superdex 16/600 75 pg column attached to the ÄKTA system filled with SEC buffer (50 mM Tris, 200 mM NaCl, and 5% glycerol, pH 8.0 at 4°C). The chromatograms from the FPLC are available in the Supplementary Information, Figure S7. Purity of fractions was verified with SDS-PAGE. Purified proteins were aliquoted and stored at -80 °C until further use.

Crystallization, X-ray measurements, and structure determination Crystallization attempts were performed using commercially available crystallization screens (*Helix*, *JCSG-plus ECO*, *Midas*, *Morpheus*; Molecular Dimensions) and multiple protein concentrations. All the protein crystallization experiments were performed on the SWISSCI MRC 2-Drop plates by the sitting drop vapor diffusion method. Crystals were transferred to cryo solution (20% ethylene glycol for 9RDS and 9QIX and 30% glycerol for 9SOJ) 1:1 with the crystallization condition and flash-cooled in liquid nitrogen.

The synchrotron data for proteins 9RDS and 9QIX were collected at beamline P13 operated by EMBL Hamburg at the PETRA III storage ring (DESY, Hamburg, Germany)(11). Data collection for protein 9SOJ was carried out at beamline 14.1 at the BESSY II electron storage ring operated by the Helmholtz-Zentrum Berlin für Materialien und Energie(12, 13).

Satisfactory diffraction results were obtained from crystals grown in the following crystallization conditions: 0.1 M sodium acetate pH 4.5, 0.2 M Li_2SO_4, 30% w/v PEG 8000 (PDB ID: 9RDS); 0.1 M sodium acetate pH 5.0, 0.05 M LiCl, 15% v/v PEG 400 (PDB ID: 9QIX); 0.1M bicine pH 9.0, 25% w/v PEG 3350 (PDB ID: 9SOJ). Data were processed with XDS implemented in XDSAPP 3.1.9 (14–20). The crystal structure was solved by molecular replacement with Phaser-MR implemented in Phenix (21) using the predicted structure from AlphaFold 2 (22–24) as a search model. The protein models were refined with phenix.refine (Phenix 1.21) (25), followed by manual inspection and building in Coot 0.9.8 (26). TLS parameters were used at the final stages of the refinement for 9QIX and 9SOJ structures (27–29). The quality of the structures was validated with the use of the MolProbity server (30) and wwPDB Validation System.

Far-UV circular dichroism experiments Far-UV circular dichroism (CD) measurements were conducted using a BioLogic MOS-450/AF-CD Spectrometer with a 0.1 mm quartz glass cuvette (106-QS, HP Quartz Glass by Hellma Analytics). Protein samples (50 μM) were prepared in a buffer containing 50 mM Tris and 200 mM NaCl (no glycerol). Measurements were carried out at room temperature, with samples kept on ice prior to measurement (with the exception of samples in urea above 7 M and guanidinium chloride to avoid crystallization). The far-UV spectrum (210–250 nm) was used, as signal reliability below 210 nm was uncertain because of the sample density. Each sample was measured independently twice with a five second acquisition period, and blanks were measured under identical conditions.

Urea solutions were freshly prepared for denaturation studies. Two preliminary experiments were performed to optimize protein concentration and incubation time. Reliable spectra were obtained at 50 μM without detector saturation, while higher concentrations showed no change in the signal. After incubation, spectra were measured across increasing urea concentrations (1 M increments) over seven hours, starting immediately after mixing. Equilibration times for the final denaturation curve were set to three hours for native protein and up to five hours in 7 M urea, at which signal change reached a plateau. Samples in concentration of 5 μM were performed in the same conditions with measurements performed on 1 mm cuvette on a Chirascan (Applied Photophysics) spectrometer.

For the refolding curve, samples were incubated in 7 M urea for 5 hours to avoid prolonged exposure to urea that would potentially decrease the refolding rate (31), before dilution to 50 μM protein and target urea concentrations. Refolding samples equilibrated for 2 hours in 7 M urea and up to 5 hours in 1.5 M urea samples. Data were averaged across repetitions and smoothed using the Savitzky-Golay algorithm.

Similarly, freshly prepared guanidinium chloride were used to perform additional tests with an incubation time following the setup for urea.

SDS-PAGE and native-PAGE gels Native proteins were analyzed by SDS-PAGE to determine their purity and molecular weight. Using the standard protocol, the samples were denatured with SDS sample buffer, heated to 95 °C for 15 min, and then transferred to SDS-PAGE containing 15% polyacrylamide gel.

To confirm that proteins with PDB IDs: 9RDS and 9QIX after denaturation will refold to the same oligomeric form, the native-PAGE electrophoresis was performed. The denaturation and refolding processes follow the same approach as in the far-UV CD experiment. Proteins were incubated in 7 M of urea with and without DTT and then diluted. Proteins were run on 12% native gel using Tris-glycine running buffer (25 mM Tris, 192 mM glycine, pH 8.3). Native-PAGE gels were run on ice, at 110 V, for three hours.

Calibration curve To estimate the molecular weight (MW) of target proteins using size-exclusion chromatography, a calibration curve was first generated using a set of standard proteins of known molecular weights. These standards were run under the same chromatographic conditions, and their elution volumes (V_e) were verified.

The partition coefficient (K_{av}) for each standard was calculated using the equation:

$$K_{\text{av}} = \frac{V_e - V_0}{V_t - V_0}$$

with V_e is the elution volume of the protein, V_0 is the void volume (Blue Dextran), and V_t is the column volume.

A calibration curve was calculated by plotting the logarithm of the molecular weight (log(MW)) of each standard protein against its corresponding K_{av}. A linear regression was performed on this plot to obtain a best-fit line, described by the equation:

$$log(MW) = xK_{av} + b$$

The regression equation was used to estimate the logarithm of the molecular weight and its antilog to estimate the molecular weight.

Size-exclusion chromatography with multi-angle light scattering (SEC-MALS) The oligomeric states and absolute molecular weights of native and refolded proteins were determined using size-exclusion chromatography with multi-angle light scattering (SEC-MALS). Samples of native and refolded proteins at a concentration of 2 mg/ml were loaded onto a Superdex 200 Increase 10/300 GL column (Cytiva) pre-equilibrated with SEC buffer at a flow rate of 0.75 ml/min using an ÄKTA Pure chromatography system (Cytiva). The eluent was passed in-line through a multi-angle light scattering detector (light scattering instrument: DAWN HELEOS 8 2022) operating at a wavelength of 658 nm, a refractive index detector (Optilab rEX) at 1.340, and a calibration constant at 3.3705×10^{-5} [1/(V·cm)]. Data acquisition and analysis were performed using ASTRA 6 software (Wyatt Technology) and Python codes.

The differential refractive index increment (dn/dc) value used for molecular weight determination was assumed to be 0.185 ml/g, a standard value for proteins. The weight-averaged molecular weight (MW) was calculated across the peak using the Zimm model. The protein concentration was determined from the UV absorbance at 280 nm and the refractive index signal. The light scattering (LS), ultraviolet absorbance (UV), and differential refractive index (dRI) signals were recorded and analyzed to determine the molecular weight and assess the oligomeric state of the protein. The molecular weights obtained from SEC-MALS were compared with those estimated from the SDS-PAGE, native-PAGE, and SEC analyses to confirm the oligomeric state.

The MW can be calculated directly from first principles using the equation:

$$MW = \frac{R(0)}{Kc\left(\frac{dn}{dc}\right)^2}$$

where the reduced Rayleigh ratio, R(0), represents the intensity of light scattered by the analyte relative to the incident laser intensity, extrapolated to zero scattering angle, K is the optical constant, incorporates system parameters such as the wavelength of the laser and the refractive index of the solvent, c is the concentration determined from either UV at 280 nm or the dRI signal, and dn/dc is refractive index increment, which reflects the change in refractive index between the analyte and the buffer (32).

Analytical size-exclusion chromatography (SEC) Analytical size-exclusion chromatography (SEC) was performed using the NGC chromatography FPLC System (Bio-Rad) with a Superdex 200 Increase 10/300 GL column (Cytvia). Protein samples were loaded into a 500 μl injection loop and then separated at a flow rate of 0.65 ml/min. Analyzed proteins in their native state and after re-folding (same setup as for the far-UV CD experiment) were used at a concentration of 40 μM for proteins with PDB IDs: 9RDS and 9QIX, in the SEC buffer used for purification. Calibration of the Superdex 200 column was performed with a Gel Filtration Markers Kit (MWGF70, Sigma-Aldrich) according to the manufacturer's instructions.

Molecular Dynamics simulations The all-atom MD simulations were performed using the GROMACS 2023.1 package with the CHARMM36 force field. The starting conformations for the proteins are based on the crystal structures (PDB id: 9RDS and 9QIX), which were simulated in oligomeric states present in crystals: monomer in the case of 9RDS and dimer for 9QIX. The proteins were solvated in a dodecahedron box with a 12 Å buffer of TIP3P explicit water molecules. Ions were added to neutralize the system and mimic physiological concentration (100 mM). The system was first minimized using the steepest descent algorithm. This was followed by a 100 ps NVT equilibration phase, and then a 500 ps NPT equilibration phase to stabilize the system's temperature and pressure. The temperature was maintained at 298 K using the V-rescale thermostat, and the pressure was controlled at 1 bar using the C-rescale barostat. Production MD runs were carried out for 200 ns under the NPT ensemble. A 2 fs timestep was used for the integration of the equations of motion. The LINCS algorithm was applied to constrain all bonds involving hydrogen atoms. Short-range electrostatic and van der Waals interactions were calculated using a cutoff of 12 Å, while long-range electrostatic interactions were treated with the Particle Mesh Ewald (PME) method. Trajectories were saved every 10 ps for subsequent analysis.

To simulate protein folding and unfolding, we employed a C_α coarse-grained structure-based model representation (33) based on the SMOG server (34). We use the shadow native contact map (35); we consider a native contact as formed when the distance between a pair of C_α atoms is less than 1.2 times their native distance, and we mimic these interactions by the Gaussian potential (36). All simulations were conducted in Gromacs v4.5.4 using leap-frog stochastic dynamics with an inverse friction coefficient equal to 1.0 and with a time step equal to 0.0005 τ. First, we performed individual long thermal unfolding trajectories at T=180 ϵ/k_B in order to generate a large number of unfolded and unknotted conformations. Next, 300 folding trajectories for each protein were executed at T=128 ϵ/k_B and they started from randomly chosen topologically trivial and unfolded conformations.

The unfolding process was studied at a temperature equal to 140.0 ϵ/k_B. The protein starts to unfold at the N-terminal end where it sequentially detaches consecutive layers until the chain is left with a knot at the native position. The layers unfold quickly one after another and it lasts around 100 τ. It takes more time to unknot the unfolded protein chain and the unknotting times are of the order of magnitude of 1000 τ.

Evolutionary analysis For each representative from sequential clustering (Figure S11) and Knotted Solenoid 9RDS, a structural alignment was done for the Knotted Solenoid part of the structure. Each structure was then split into 5 parts (Figure S16), in order: N-terminal section and first two loops, skipping loop, backtracking loop, threading loop, and C-terminal section. New sequential constructs have been then done for each protein: first with only the removed backtracking loop, and second with the removed backtracking loop and the reversed order of loops. All constructs were then used as input to BLASTP against the full UniProt Database in order to detect if such an operation would allow for the detection of even distant homologs related to Knotted Solenoids, but potentially without "skip-and-backtrack" shift and trivial topology. However, the results have shown that all proteins were either from the Knotted Solenoid group or without AlphaFold prediction, yet still aligned closely to the native protein (Figure S16). This suggests that the unique skip-and-backtrack coil topology arose independently rather than by simple domain rearrangement.

Detection of a unique set of structural features of Knotted Solenoids. Knotted Solenoids constitute a subgroup within the broader class of β-solenoids and are classified under CATH lineage 2.160. To identify structural features distinguishing Knotted Solenoids from other members of this class, we performed a comprehensive analysis of all biological assemblies deposited in the RCSB PDB and assigned to CATH lineage 2.160, including all 2.160.X.Y subgroups (database accessed 20.02.2026). No additional filtering of assemblies was applied as to avoid exclusion of structural variants. Thus we acknowledge that some assemblies might include crystal packing contacts, which however will not influence the final conclusion of the analysis.

The main β-solenoid core was defined in two stages. First, domain boundaries were extracted according to CATH annotations. Second, consistency with the characteristic axial rise of β-solenoids described by Kajava and Steven (37) was evaluated using a geometric distance-based criterion (around 4.8 Å of axial rise). For each Cα atom within a CATH domain, neighboring Cα atoms located at a distance of 4.5–5.1 Å were identified. To prevent detection of local backbone contacts, residues within ± 5 sequence positions were excluded from the search. Continuous solenoidal segments were defined as stretches of at least five marked residues, allowing gaps of up to two consecutive unmarked residues to reduce overall noise and excessive segmentation while not impacting the quality of detection.

The second part of the analysis addressed oligomeric organization, with particular emphasis on dimeric assemblies. Inter-chain spatial proximity was assessed by identifying Cα pairs from different chains located within 8 Å. This analysis was used solely as a geometric descriptor of inter-chain proximity rather than as a formal definition of a biological interface. Each domain was divided into three regions: the N-terminal 20%, the central 60%, and the C-terminal 20%. This allowed distinction between predominantly lateral (central-region) and terminal (end-to-end) chain arrangements.

All dimeric assemblies were subsequently examined manually. During this inspection, we evaluted a number of features including: the cross-sectional geometry of the solenoidal channel (e.g., triangular, rectangular, or horseshoe-shaped), the relative orientation of chains (parallel or antiparallel), the alignment order (e.g., N–C/N–C versus N–C/C–N), overall symmetry, and the hydrophobic character of the inter-chain contact surface. This combined automated and manual assessment enabled the identification of feature combinations unique to Knotted Solenoids within the analyzed β-solenoid dataset.

References

1. W Niemyska, et al., AlphaKnot: server to analyze entanglement in structures predicted by AlphaFold methods. *Nucleic Acids Res.* **50**, W44–W50 (2022).
2. P Rubach, M Sikora, AI Jarmolinska, AP Perlinska, JI Sulkowska, AlphaKnot 2.0: a web server for the visualization of proteins' knotting and a database of knotted AlphaFold-predicted models. *Nucleic Acids Res.* **52**, W187–W193 (2024).
3. G Torres, On the alexander polynomial. *Annals Math.* **57**, 57–89 (1953).
4. P Dabrowski-Tumanski, P Rubach, W Niemyska, BA Gren, JI Sulkowska, Topoly: Python package to analyze topology of polymers. *Briefings Bioinforma.* **22**, bbaa196 (2021).
5. M Van Kempen, et al., Fast and accurate protein structure search with Foldseek. *Nat. Biotechnol.* **42**, 243–246 (2024).
6. F Gabler, et al., Protein sequence analysis using the MPI bioinformatics toolkit. *Curr. Protoc. Bioinforma.* **72**, e108 (2020).
7. RD Finn, J Clements, SR Eddy, HMMER web server: interactive sequence similarity searching. *Nucleic Acids Res.* **39**, W29–W37 (2011).
8. K Katoh, J Rozewicki, KD Yamada, MAFFT online service: multiple sequence alignment, interactive sequence choice and visualization. *Briefings bioinformatics* **20**, 1160–1166 (2019).
9. GE Crooks, G Hon, JM Chandonia, SE Brenner, WebLogo: a sequence logo generator. *Genome Res.* **14**, 1188–1190 (2004).
10. TL Bailey, J Johnson, CE Grant, WS Noble, The MEME suite. *Nucleic Acids Res.* **43**, W39–W49 (2015).
11. M Cianci, et al., P13, the embl macromolecular crystallography beamline at the low-emittance petra iii ring for high-and low-energy phasing with variable beam focusing. *J. Synchrotron Radiat.* **24**, 323–332 (2017).
12. M Gerlach, U Mueller, MS Weiss, The mx beamlines bl14. 1-3 at bessy ii. *J. Large-scale Res. Facil. JLSRF* **2**, A47–A47 (2016).

13. U Mueller, et al., The macromolecular crystallography beamlines of the Helmholtz-Zentrum Berlin at the BESSY II storage ring: history, current status and future directions. *Synchrotron Radiat.* **32** (2025).
14. M Krug, MS Weiss, U Heinemann, U Mueller, Xdsapp: a graphical user interface for the convenient processing of diffraction data using xds. *J. Appl. Crystallogr.* **45**, 568–572 (2012).
15. KM Sparta, M Krug, U Heinemann, U Mueller, MS Weiss, Xdsapp2. 0. *J. Appl. Crystallogr.* **49**, 1085–1092 (2016).
16. AA Vaguine, J Richelle, S Wodak, Sfcheck: a unified set of procedures for evaluating the quality of macromolecular structure-factor data and their agreement with the atomic model. *Biol. Crystallogr.* **55**, 191–205 (1999).
17. W Kabsch, xds. *Acta Crystallogr. Sect. D: Biol. Crystallogr.* **66**, 125–132 (2010).
18. K Diederichs, Some aspects of quantitative analysis and correction of radiation damage. *Biol. Crystallogr.* **62**, 96–101 (2006).
19. P Evans, Scaling and assessment of data quality. *Biol. Crystallogr.* **62**, 72–82 (2006).
20. PD Adams, et al., Phenix: a comprehensive python-based system for macromolecular structure solution. *Acta Crystallogr. Sect. D: Biol. Crystallogr.* **66**, 213–221 (2010).
21. AJ McCoy, et al., Phaser crystallographic software. *Appl. Crystallogr.* **40**, 658–674 (2007).
22. MA Brems, R Runkel, TO Yeates, P Virnau, Alphafold predicts the most complex protein knot and composite protein knots. *Protein Sci.* **31**, e4380 (2022).
23. P Dabrowski-Tumanski, A Stasiak, Alphafold blindness to topological barriers affects its ability to correctly predict proteins' topology. *Molecules* **28**, 7462 (2023).
24. AP Perlinska, et al., AlphaFold predicts novel human proteins with knots. *Protein Sci.* **32**, e4631 (2023).
25. D Liebschner, et al., Macromolecular structure determination using x-rays, neutrons and electrons: recent developments in phenix. *Acta Crystallogr. Sect. D: Struct. Biol.* **75**, 861–877 (2019).
26. P Emsley, B Lohkamp, WG Scott, K Cowtan, Features and development of coot. *Acta Crystallogr. Sect. D: Biol. Crystallogr.* **66**, 486–501 (2010).
27. EA Merritt, Expanding the model: anisotropic displacement parameters in protein structure refinement. *Biol. Crystallogr.* **55**, 1109–1117 (1999).
28. F Zucker, P Champ, EA Merritt, Validation of crystallographic models containing tls or other descriptions of anisotropy. *Biol. Crystallogr.* **66**, 889–900 (2010).
29. EA Merritt, To b or not to b: a question of resolution? *Biol. Crystallogr.* **68**, 468–477 (2012).
30. CJ Williams, et al., Molprobity: more and better reference data for improved all-atom structure validation. *Protein Sci.* **27**, 293–315 (2018).
31. KM Kathir, TKS Kumar, D Rajalingam, C Yu, Time-dependent changes in the denatured state (s) influence the folding mechanism of an all β-sheet protein. *J. Biol. Chem.* **280**, 29682–29688 (2005).
32. D Some, H Amartely, A Tsadok, M Lebendiker, Characterization of proteins by size-exclusion chromatography coupled to multi-angle light scattering (sec-mals). *J. Vis. Exp* **148**, e59615 (2019).
33. C Clementi, H Nymeyer, JN Onuchic, Topological and energetic factors: what determines the structural details of the transition state ensemble and "en-route" intermediates for protein folding? An investigation for small globular proteins. *J. Mol. Biol.* **298**, 937–953 (2000).
34. JK Noel, PC Whitford, KY Sanbonmatsu, JN Onuchic, SMOG@ ctbp: simplified deployment of structure-based models in GROMACS. *Nucleic Acids Res.* **38**, W657–W661 (2010).
35. PC Whitford, et al., An all-atom structure-based potential for proteins: bridging minimal models with all-atom empirical forcefields. *Proteins: Struct. Funct. Bioinforma.* **75**, 430–441 (2009).
36. H Lammert, A Schug, JN Onuchic, Robustness and generalization of structure-based models for protein folding and function. *Proteins: Struct. Funct. Bioinforma.* **77**, 881–891 (2009).
37. AV Kajava, AC Steven, β-rolls, β-helices, and other β-solenoid proteins. *Adv. Protein Chem.* **73**, 55–96 (2006).
